# Inhibition/excitation balance is associated with functional connectivity during cognitive flexibility performance

**DOI:** 10.1093/braincomms/fcag319

**Published:** 2026-09-28

**Authors:** Geraldine Rodríguez-Nieto, Mark Mikkelsen, Stephan P Swinnen

**Affiliations:** Movement Control and Neuroplasticity Research Group, Biomedical Sciences, KU Leuven, Leuven 3001, Belgium; KU Leuven Brain Institute, KU Leuven, Leuven 3000, Belgium; Rehabilitation Research Institute (REVAL), University of Hasselt, Diepenbeek 3500, Belgium; Department of Radiology, Weill Cornell Medicine, New York, NY, USA; Movement Control and Neuroplasticity Research Group, Biomedical Sciences, KU Leuven, Leuven 3001, Belgium; KU Leuven Brain Institute, KU Leuven, Leuven 3000, Belgium

**Keywords:** GABA, glutamate, functional connectivity, cognitive flexibility, ageing

## Abstract

The primary excitatory and inhibitory neurotransmitters, glutamate and GABA, regulate neural communication. In this cross-sectional study, we investigated whether the large-scale functional connectivity of two important brain hubs, the inferior frontal cortex and the inferior parietal lobe, is associated with changes in the GABA/Glx (glutamate + glutamine) balance in the in vivo human brain during a cognitive flexibility task. Furthermore, we explored age differences in this relationship, given the alterations in neurotransmitter levels and in cognitive flexibility performance in older adults. Forty young (18–35 years; 26 females) and forty older (60–85 years; 21 females) adults participated. The MRI protocol included functional MRI and baseline and task-related magnetic resonance spectroscopy. Results showed that task-induced modulation of GABA+/Glx is associated with functional connectivity from the inferior frontal cortex and inferior parietal lobe during cognitive flexibility performance. In particular, young adults showed stronger functional connectivity associated with increased inhibitory tone in the inferior frontal cortex than older adults, whereas the pattern was reversed in the inferior parietal lobe. In addition, whereas the connectivity between the left inferior parietal lobe and the bilateral putamen in young adults predicted better cognitive flexibility (left putamen: r = 0.41, *P* = 0.05; right putamen: r = 0.51 *P* = 0.01; controlling for GABA modulation), this relationship had an opposite direction in older adults (left putamen: r = −0.33, *P* = 0.05; right putamen: r = −0.31 *P* = 0.01; controlling for GABA modulation). In sum, these results show that neurometabolic dynamics and functional connectivity underlying cognitive flexibility undergo substantial changes during ageing.

## Teaser

Rodríguez Nieto *et al*. showed that changes in GABA+/Glx balance in two executive brain hubs relate to functional connectivity during cognitive flexibility performance, with important differences between young and older adults. GABA+/Glx modulation in the inferior parietal lobe and functional connectivity between this region and the putamen were related to performance quality.

## Graphical Abstract

**Graphical Abstract:**
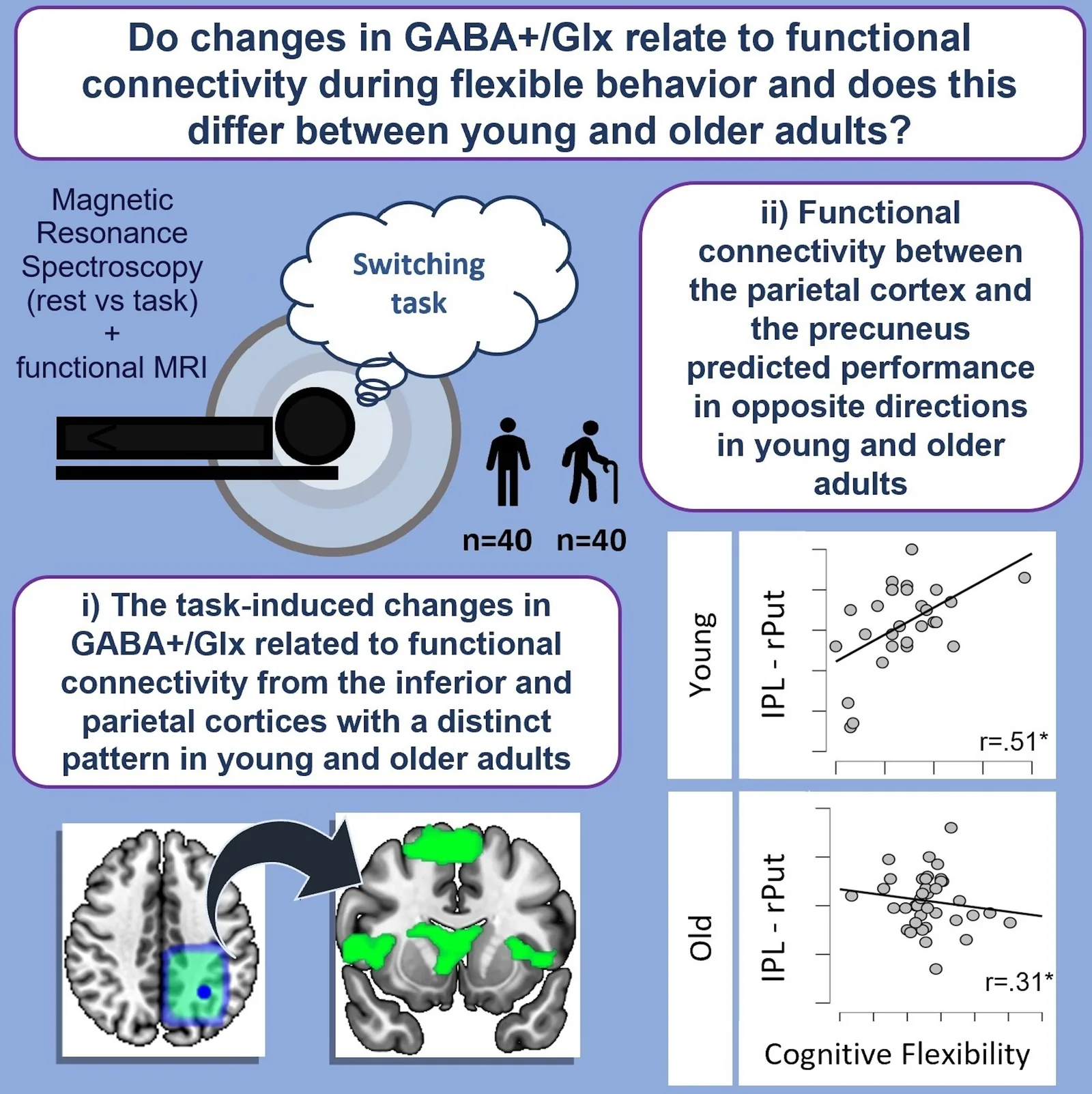

## Introduction

Cognitive flexibility is part of the executive function domain that enables humans to switch among different mental states and schemas. This ability provides an advantage in changing environments by allowing flexible adaptation.

The inferior frontal cortex (IFC), inferior parietal lobe (IPL), insula and cingulate have been shown to be consistently engaged during cognitive flexibility paradigms.^1,2^ The IFC and IPL are part of the canonical executive frontoparietal network, and as such, form part of a high-order cognitive functioning system.^3^ In addition, these two regions are considered association areas; that is, they integrate information from other specialized areas, making them functionally flexible regions that enable the execution of diverse cognitive functions.^4,5^

GABA and glutamate are the most abundant neurotransmitters in the brain, and they regulate neural activity by increasing (glutamate) or decreasing (GABA) the excitability of the neurons. Magnetic resonance spectroscopy (MRS) can noninvasively quantify the concentration of these neurometabolites in specific brain regions.

The relationship between GABA and glutamate + glutamine (Glx) levels and local BOLD response has been studied at baseline and during experimental paradigms.^6-9^ These studies have shown that a decreased inhibitory tone is related to a higher local BOLD response in visual, emotional and executive paradigms.

Because GABA and Glx modulate the activity of neural populations supported by large-scale connections, we expect GABA and Glx levels in association areas (or richly connected regions) to be associated with functional connectivity. Few studies have shown that baseline GABA and Glx relate to functional connectivity during different memory paradigms.^10-12^ To our knowledge, the relationship between functional connectivity and the modulation of neurometabolites has not been investigated.

Along these lines, we expect the inhibitory/excitatory (GABA/Glx) balance in the IFC and IPL (nodes of the executive network and richly connected areas)^2^ to be associated with functional connectivity during the performance of cognitive flexibility tasks, which requires accurate and flexible filtering of information, involving the alternating selective activation of neural networks.

Previous studies have shown age-related differences in the relationship between neurometabolites and executive function performance. One study showed that reduced levels of GABA+ (GABA plus macromolecules) in the sensorimotor cortex of older adults -but not of young adults- were related to slower stopping in an inhibition task.^13^ In our previous report, we showed that the modulation of the GABA+/Glx balance in the inferior parietal cortex was related to cognitive flexibility performance. More specifically, a decrease from baseline to task was related to better performance in young adults, while the opposite pattern was observed in older adults.^9^ Finally, an EEG-MRS study suggested that GABA+ levels in the sensorimotor cortex underlie adjustments in interhemispheric connectivity to uphold cognitive flexibility in older adults.^14^ In addition, young and older adults show differences in the functional connectivity and brain network dynamics associated with executive processes.^5,15-17^ Therefore, we expect the role of GABA/Glx in functional connectivity during executive processing to differ between young and older individuals.

In sum, we aim to investigate functional connectivity patterns within two important executive hubs, IFC and IPL, during cognitive flexibility in young and older adults. As the IFC and IPL are association areas with rich connections that support executive performance, we expect that the inhibition/excitation balance modulation resulting from cognitive flexibility performance in these regions will be related to functional connectivity. Furthermore, we expect that the degree of functional connectivity will be relevant to the performance level of cognitive flexibility tasks, under the assumption that the IFC and IPL integrate and coordinate information from distant brain regions. Finally, we expect the relationships among inhibitory/excitatory balance, functional connectivity and behaviour to differ between young and older adults.

## Materials and methods

This study was carried out in accordance with the methods detailed previously in Rodríguez-Nieto *et al*., 2024.

### Participants

40 young and 40 older healthy adult participants were recruited for this study in Leuven, Belgium in 2023 (young: mean age = 23.38 SD = 3.81, range = 18–35 years old, 26 female; old: mean ± SD age = 69.52, SD = 5.42, range = 60–85 years old, 21 female). The sample size was defined considering previous literature.^10-12^ All participants reported the absence of psychiatric or neurological disorders and were assessed with the Montreal Cognitive Assessment (MoCA). All participants scored above the cutoff point (young: mean score = 27.4, range = 24–30; old: mean score = 27.1, range = 22–30),^18^ except one older adult who was excluded for this reason (score = 22). Participants filled the Edinburgh Handedness Inventory. 37 young adults and 37 older adults were right-handed; three younger adults and two older adults were left-handed, and one older adult was ambidextrous. This proportion is similar to the reported proportion for the general population.^19^ The experiment was evaluated and approved by the KU Leuven Ethical Committee (protocol number: s61577), and all participants provided their written informed consent. The data from the present study have already been used to investigate the relationship between neurometabolic modulation (ΔGABA+/Glx) and cognitive flexibility in young and older adults.^9^

### Procedure

**Figure 1:**
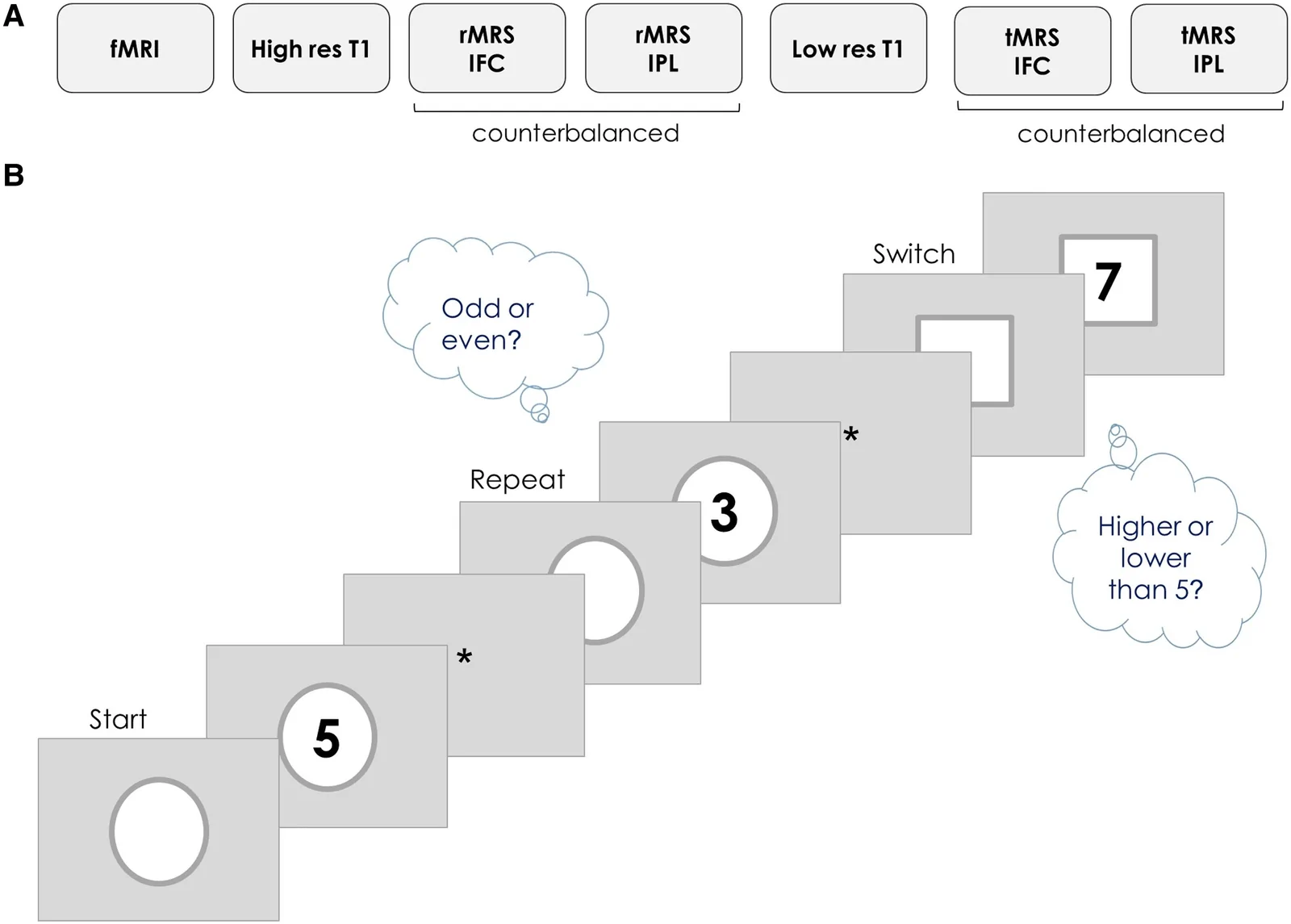
**Study design.** (**A**) Magnetic resonance scan protocol. High res T1: High resolution T1; rMRS: resting state MRS; Low res T1: Low resolution T1; tMRS: task-related MRS during cognitive flexibility task performance. (**B**) Cognitive flexibility task design. Individuals followed one of two rules: decide whether the number was odd or even, or whether it was higher or lower than 5, based on the shape surrounding the number. If the rule was the same as in the previous trial, this was a Repeat trial; if it was different, this was a Switch trial. The performance of Switch and Repeat trials was compared to generate a cognitive flexibility index.

Before entering the MRI room, participants completed 16 practice trials of the cognitive flexibility task (Rule-switching task). During the MRI session, a high-resolution *T*_1_-weighted structural scan was acquired, and participants performed the task during fMRI data collection and during half of the MRS blocks. Two MRS scans at baseline (resting-state MRS: rMRS) with the voxels positioned at the IFC and IPL were collected (counterbalanced across participants). This was followed by a brief *T*_1_-weighted structural scan aimed at minimizing the impact of intermediate participant movement on voxel location by utilizing it for image co-registration of the subsequent scans. Last, two MRS scans with the voxels positioned in the IFC and IPL (counterbalanced across participants) while participants executed the task (task-related MRS: tMRS; Fig. 1A) were collected.

### Behavioural task

In the rule-switching flexibility task (adapted^20^), participants utilized a button box to respond to stimuli displayed on the screen. They indicated whether a displayed number was lower or higher than five (left or right button press) when a cue preceded the number and simultaneously enclosed by a circle. Additionally, they determined whether the number was odd or even (left or right button press) when preceded by a cue and enclosed by a square (Fig. 1B). The task included two types of trials: repeat trials, in which the rule remained the same as in the previous trial, and switch trials, in which the rule changed. The sequence of trials was pseudo-randomized.

During the fMRI collection, participants completed 94 trials, with a cue duration of 300 ms, cue + stimulus duration of 1700 ms, and inter-trial interval (ITI) ranging from 3 to 4 s. In each of the two MRS blocks, participants completed 132 trials, with a cue-alone duration of 400 ms, a cue + stimulus duration of 2.2 s, and an ITI ranging from 2 to 4 s. The task was implemented using PsychoPy.^21^

A cognitive flexibility index was generated by subtracting the number of hits in repeat trials from the number of hits in switch trials. To avoid the effects of learning, only the trials from the fMRI block were considered for this calculation. A higher index indicated better cognitive flexibility performance.

### Imaging data acquisition

MRI data were collected using a Philips Achieva 3T scanner equipped with a 32-channel head coil. For the main anatomical scan, a high-resolution 3D *T*_1_-weighted image was obtained using the MPRAGE sequence (TE/TR/TI = 4.6/9.6/900 ms; voxel size = 0.98 × 0.98 × 1.2 mm^3^; FOV = 192 × 250 × 250; 160 coronal slices; scan time: ∼7 min). An intermediate short *T*_1_-weighted structural image was also acquired to ensure correct alignment of MRS voxels.

Functional scans (fMRI) were acquired using an ascending gradient EPI sequence for *T*_2_*-weighted images (TE/TR = 33/1000 ms; flip angle = 70°; 42 transverse slices; interslice gap = 0.5 mm; voxel size = 2.15 × 2.14 × 3.0 mm^3^; FOV = 240 × 240 × 146.5 mm^3^).

For MRS, a Hadamard Encoding and Reconstruction of MEGA-Edited Spectroscopy (HERMES) sequence was employed (TE/TR = 80/2000 ms; 5 kHz spectral width; 4096 datapoints; 320 water-suppressed transients; 16 unsuppressed transients; total scan time: ∼12 min.^22^ Two baseline and two task-related MRS sequences were used to measure GABA+ and Glx levels in the left IFC (4 × 2.5 × 2.5 cm^3^ voxel) and in the left IPL (3 × 3 × 3 cm^3^ voxel). The voxels were selected and positioned according to a meta-analysis of the neural substrates of cognitive flexibility and the rule-switching task.^2^

To position the voxel in the left IFC, the next procedure was followed: a) centre the voxel in the first axial slice above the ventricles, at one third of the sagittal midline of the left hemisphere, b) align the centre length of the voxel parallel to the IFC with three-quarters of its length over the inferior frontal junction from the sagittal view, and c) position the top of the voxel parallel to the cortical surface from the coronal view, with adjustments made to avoid covering the ventricles or subarachnoid space if necessary.

For the left IPL voxel, the procedure was as follows: a) locate the voxel four slices above the last axial view of the ventricles and the first view after the ventricles in the coronal plane, b) align one side parallel to the occipito-parietal sulcus, c) move the voxel towards the axial midline of the left hemisphere, and d) in the sagittal plane, align it parallel to the IPL, ensuring adequate coverage.

### fMRI data analysis

fMRI data were analyzed with FSL v.6.05.^23^ Initially, BET was applied to the *T*_1_-weighted structural images to isolate the brain from the dura and skull. Subsequent preprocessing of the fMRI data included: high-pass filtering with a cut-off time constant of 90 s, motion correction using MCFLIRT, and spatial smoothing with a full-width at half-maximum (FWHM) of 8 mm. The co-registration of the functional and structural images was conducted with a linear transformation (12 degrees of freedom) and underwent visual inspection. The resulting images were normalized to the MNI152 template using linear registration (12 degrees of freedom). Data from six young participants were excluded due to technical issues and head motion.

A fixed-effects general linear model (GLM) was applied to the data of each participant, including Repeat trials, Switch trials, and inter-trial intervals as regressors along with their first temporal derivative. A *z*-threshold of >2.3 and a corrected cluster significance threshold of *P* = 0.05 were used to generate statistical maps. To calculate the mean BOLD activity for the Switch > Repeat contrast within each group, a random-effects model was employed. The results of these models were used for group comparisons (older adults > young adults and young adults > older adults; cluster-corrected maps, threshold: *P* = 0.05; *z*-values > 2.7).

### PPI

**Figure 2:**
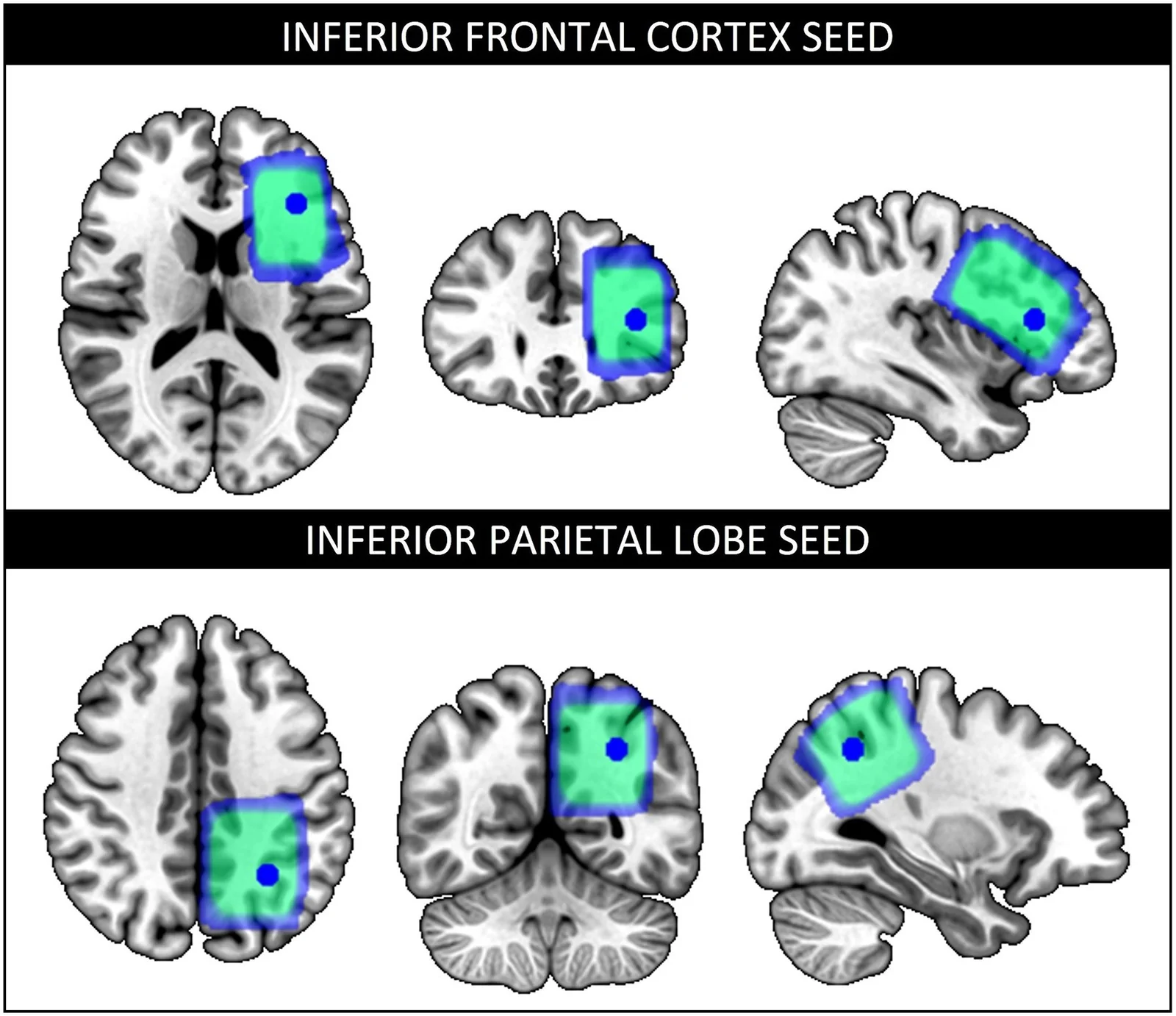
**Functional connectivity seeds.** Localization of the seeds for the functional connectivity analysis (psychophysiological interaction) in the IFC (x,y,z = −37,28,15) and in the IPL (x,y,z = −29, −53,40) and the visualization of the respective MRS voxels.

Functional connectivity was measured using psychophysiological interaction (PPI) analyses^24^ in FSL. The selection for the location of the seeds was based on two criteria: a) high BOLD activation in both groups and b) being centrally positioned within the MRS voxel. The seed for the IPL was located at x = −29, y = −53, z = 40 in MNI coordinates, based on a BOLD activation z-threshold of 4.9 for young adults and 5.2 for older adults. For IFC, the seed was located at x = −37, y = 28, z = 15 in MNI coordinates, based on a group-average BOLD activation threshold of 3.8 for young adults and 3.7 for older adults. These seeds also lie under the neural map of the Rule-Switching task reported in our previous meta-analysis.^2^ Each seed was generated with a spherical mask (27 cm^3^ voxel; Fig. 2). PPI analyses were performed to analyze the whole-brain functional connectivity during switching trials (Switch > Repeat) in young adults, older adults, and the full sample. Further PPI analyses were performed, adding the change in GABA+/Glx (ΔGABA+/Glx) as a covariate for cognitive flexibility performance to examine its positive or negative effects. The PPI group statistical maps were rendered using a *z*-threshold of >2.3 and a corrected cluster significance threshold of *P* = 0.05.

### MRS data analysis

The data were analysed with Gannet v.3.3.0,^25^ which comprised line broadening (3 Hz), eddy-current correction, and robust spectral registration.^26^ The water signal was fitted using a Lorentz-Gaussian model, and it was used as the reference metabolite. All preprocessed data were visually inspected to detect signal artefacts and assess data quality. A full report of the minimal reporting standards (MRSinMRS) as recommended by Lin *et al*.^27^ is displayed in Supplementary Table 1. GABA+ (GABA plus macromolecules) estimates exhibiting a fitting error greater than 14% (3 std. from the mean) were excluded, which resulted in the exclusion of two older participants’ IFC MRS data.

**Figure 3:**
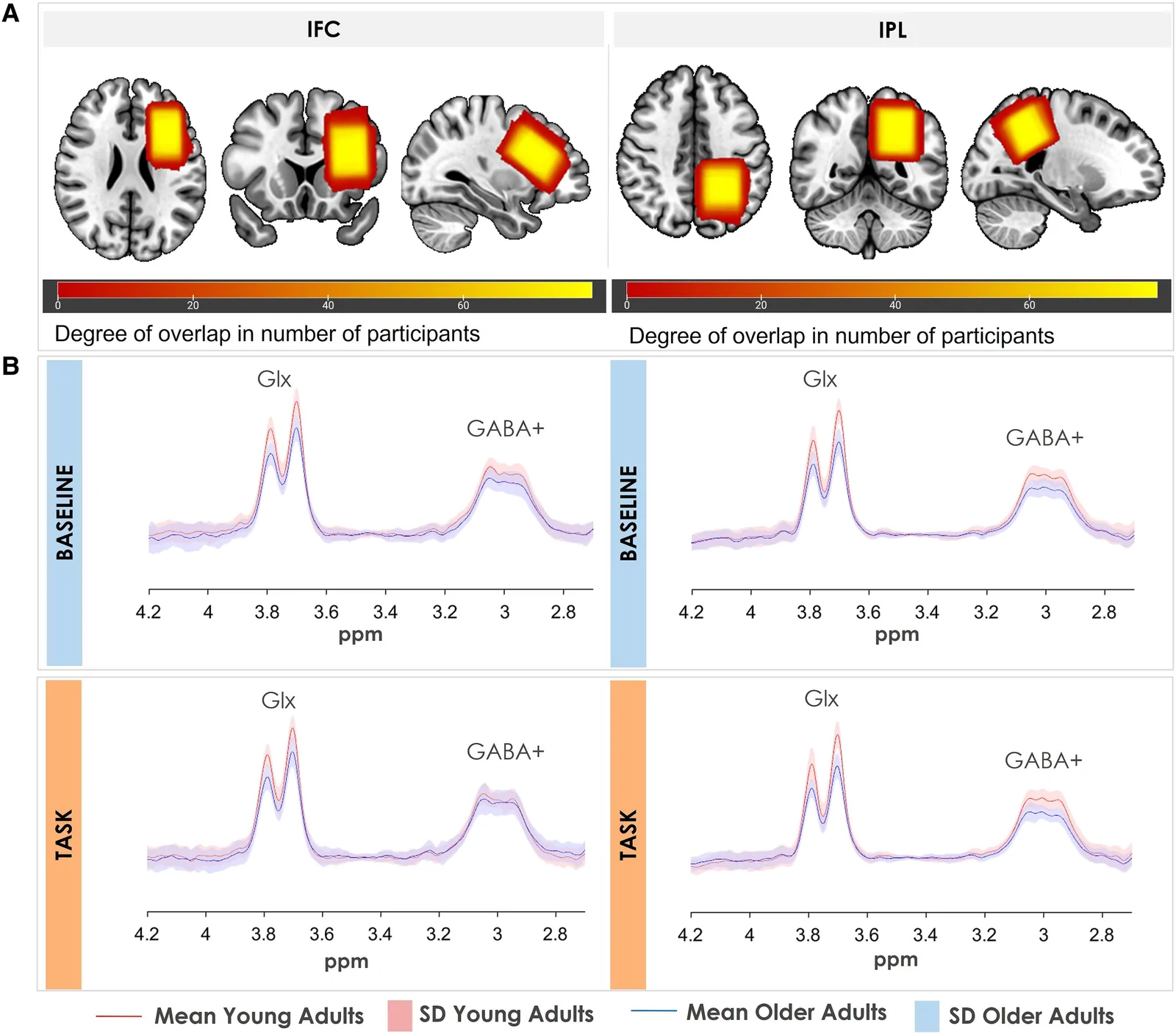
**GABA + and glx average spectra.** (A) Voxel overlap positioning from all participants (*n* = 79). (**B**) Average spectra from young and older participants during baseline (top panel) and task performance (bottom panel). In the spectra, the shaded areas indicate ± 1 std. ppm—parts per million. Young baseline IFC *n* = 40; Old baseline IFC *n* = 37; Young baseline IPL *n* = 40 Old baseline IPL *n* = 39 Young task IFC *n* = 40; Old task IFC *n* = 37; Young task IPL *n* = 40; Old task IPL *n* = 39.

Voxel masks were co-registered to their respective structural images to determine the fractions of grey matter, white matter, and cerebrospinal fluid. Metabolite levels were tissue-corrected using the α-correction method.^28^ Fig. 3 displays the overlap of IFC and IPL voxels from all participants and the average spectra for each age group at baseline and during task performance.

To indicate the task-induced change in inhibitory-excitatory balance, a GABA+/Glx modulation index (ΔGABA+/Glx) was estimated by subtracting the GABA+/Glx ratio during resting state blocks from that during the Rule-switching task performance. A positive index indicates a task-induced increase in the inhibitory tone (GABA+/Glx), while a negative index indicates a task-induced decrease.

Whereas the BOLD response and GABA+/Glx levels measured by MRS comprise different aspects of metabolic activity, in this manuscript, we will specifically refer to the MRS-derived indices (GABA+ and Glx) as metabolic activity.

### Statistical analyses

To assess the effect of age, training block and their interaction on cognitive flexibility performance, a mixed-effects repeated-measures GLM was conducted.

As an association between the inhibition/excitation modulation in IPL and cognitive flexibility performance has been reported,^9^ we conducted Pearson correlations to investigate whether the parameters from the functional connectivity maps associated with the inhibition/excitation modulation in IPL were related to cognitive flexibility performance. This analysis was performed independently for young and older adults.

Finally, we conducted mediation analyses (PROCESS software)^29^ to investigate whether the relationship between inhibition/excitation balance and cognitive flexibility performance was mediated by functional connectivity. The parameters of the functional connectivity maps were used (from the respective sample maps) for this purpose.

Further fine-grained analyses were conducted, choosing the connectivity between the IPL and the inferior frontal gyrus, putamen, and precuneus as mediators, which have been identified as core processing regions of cognitive flexibility.^2,30^ For this purpose, we extracted the beta parameters from the individual PPI maps from the mask created for each of these regions (left inferior frontal gyrus pars triangularis, left putamen, right putamen, and precuneus) from the Harvard-Oxford atlas (RRID:SCR_001476).

## Results

### Cognitive flexibility performance

A mixed-effects repeated-measures GLM was performed to assess Age, Block and Age × Block interaction effects on task performance. Younger individuals performed better overall (F_(1,71)_ =10.71, *P* = 0.002; ղ^2^ = 0.14), and the full sample showed improvement across the blocks (F_(1,71)_ = 80.55, *P* < 0.001; ղ^2^ = 0.56), but an Age × Block interaction was also observed (F_(1,71)_ = 9.87, *P* = 0.002; ղ^2^ = 0.13). Whereas young adults showed a better performance than older adults during the first and second blocks (fMRI: t_(70)_ = −3.33, *P* = <0.001, *d* = 0.79; young: mean = −7.17, SD = 8.61, old: mean = −14.31, SD = 9.22; MRS: t_(70)_ = −3.13, *P* = 0.001, *d* = 0.74; young: mean = 0.14, SD = 0.62, old: mean = −0.32 ± 0.62), young and older individuals had a similar performance during the third block (MRS: t_(70)_ = 0.26, *P* = 0.39, *d* = 0.07; young: mean = −0.58, SD = 0.68, old: mean = −0.53, SD = 0.84). As most participants reached a ceiling effect by the last block, and to exclude learning effects, we considered the cognitive flexibility index from the first block (fMRI) in further analyses, which reflects the variability in the default cognitive flexibility of participants better (Supplementary Fig. 1).

### BOLD response during cognitive flexibility

During switching trials (Switch > Repeat), both young and older adults exhibited activation in the bilateral inferior and superior parietal lobes, the presupplementary motor area (extending to the left inferior and middle frontal cortices and the insula), and the anterior cingulate cortex. Generally, the overall BOLD response in young participants was more localized to the left hemisphere compared to older adults. Statistical analysis directly comparing the two age groups revealed that older adults showed a stronger BOLD response in the bilateral IPLs and the occipital cortex (Supplementary Table 2; Supplementary Fig. 2).

### Functional connectivity

**Figure 4:**
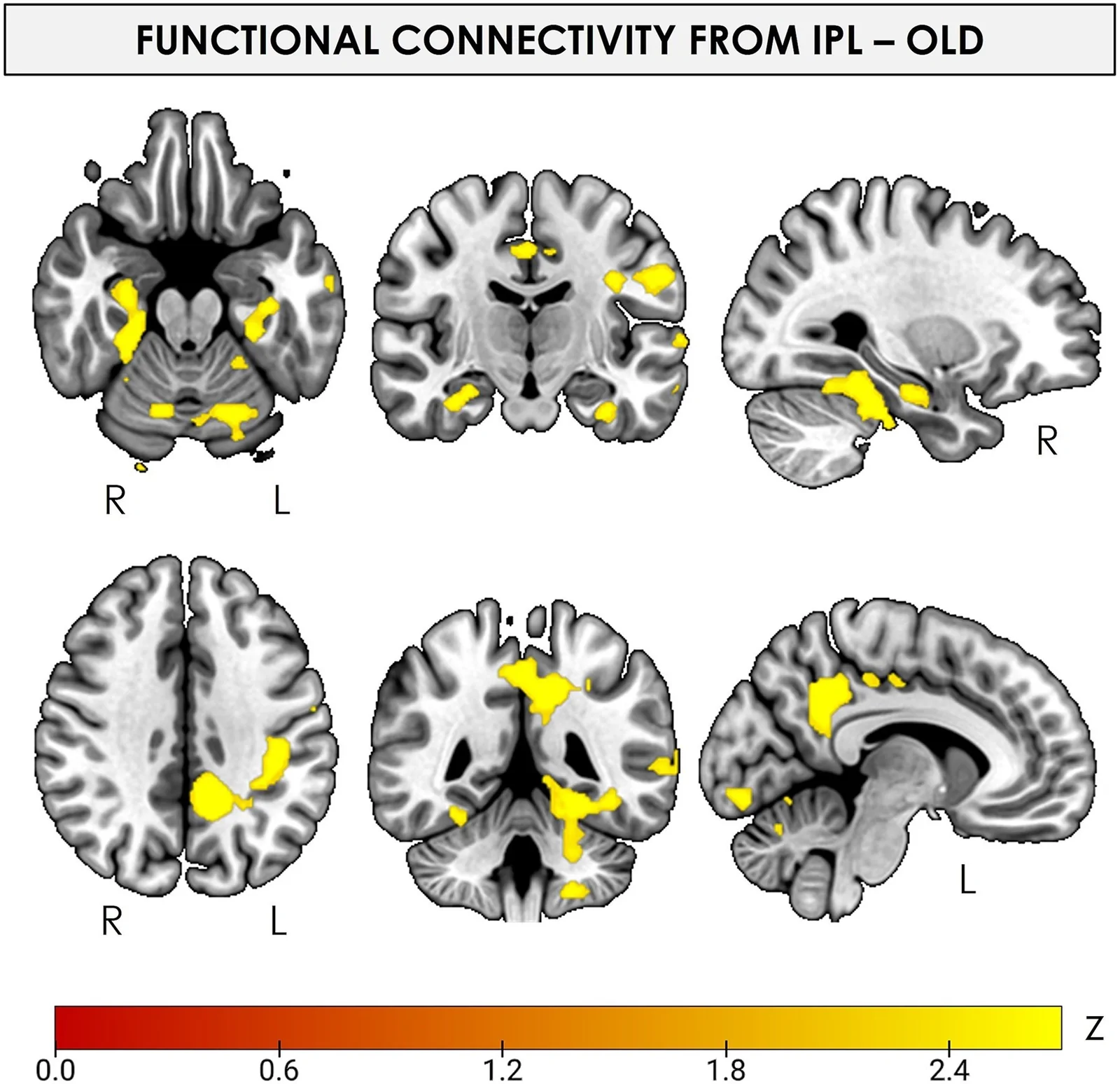
**Functional connectivity from IPL in older adults.** Functional connectivity during cognitive flexibility performance (Switch > Repeat) from the IPL seed in older adults (Psychophysiological interaction; *z* < 2.7, cluster correction *P* = 0.05, *n* = 39).

During switching trials (Switch > Repeat), no significant functional connectivity patterns were observed for the IFC in either sample. Similarly, no significant functional connectivity patterns were observed for the IPL in younger adults or in full sample. However, in older adults, the IPL seed showed a significant coupling with the precuneus, superior parietal lobe, postcentral gyrus, lingual gyrus, planum temporale, (occipital and temporo-occipital) fusiform gyrus, middle and superior temporal gyri, and cerebellum on the left hemisphere and with the bilateral precuneus. The left cerebellum cluster extended to the left cerebellum and bilaterally reached the parahippocampal gyrus and the hippocampus (Fig. 4; Supplementary Table 3). A direct statistical comparison of IPL functional connectivity between the two groups showed significantly greater connectivity from the IPL to the right occipital fusiform gyrus, cerebellum, and occipital pole in older adults than in young adults (Supplementary Table 3).

### Inhibition/excitation balance effect on functional connectivity

**Figure 5:**
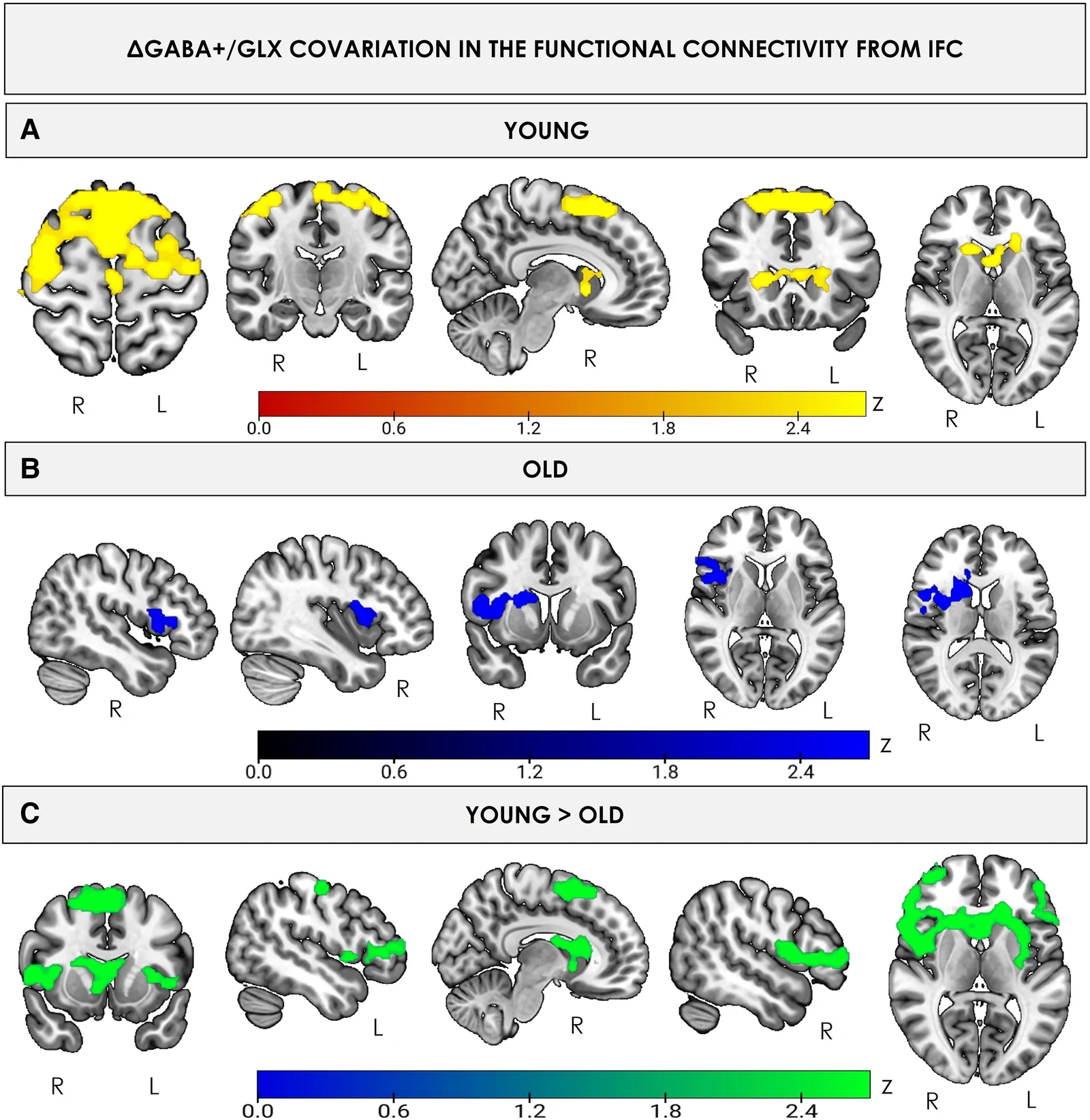
**ΔGABA+/Glx covariation effect on functional connectivity from the IFC.** (**A**) An increase in inhibition/excitation balance in the IFC during cognitive flexibility was associated with enhanced functional connectivity from this region in young adults (*n* = 34). (**B**) A decrease in inhibition/excitation balance in the IFC during cognitive flexibility was associated with negative coupling from this region in older adults (*n* = 39). (**C**) Younger adults showed higher functional connectivity from the IFC, associated with increased inhibition/excitation in this region (*n* = 73). All maps: Psychophysiological interaction—covariation effect, z = 2.7, cluster correction *P* = 0.05.

The PPI analyses with ΔGABA+/Glx as a covariate with the seed in the IFC showed a positive coupling with the bilateral superior frontal gyrus, the right precentral gyrus, and the right caudate in young adults. This effect indicated that a task-induced increase in inhibitory tone (GABA+/Glx) was associated with greater connectivity between the IFC and the aforementioned brain regions. In contrast, in older adults, a task-induced increase in GABA+/Glx was associated with a stronger negative coupling of the IFC with the insular cortex, caudate, inferior frontal gyrus (pars opercularis and pars triangularis), and the central opercular cortex in the right hemisphere. A comparison between the two groups showed increased IFC functional connectivity, bilaterally linked to ΔGABA+/Glx, in young adults in the superior frontal gyrus and the precentral gyrus, and in the central opercular cortex, the frontal pole, and the supplementary cortex in the right hemisphere (Supplementary Table 4; Fig. 5). No significant differences were found in the Old > Young comparison.

**Figure 6:**
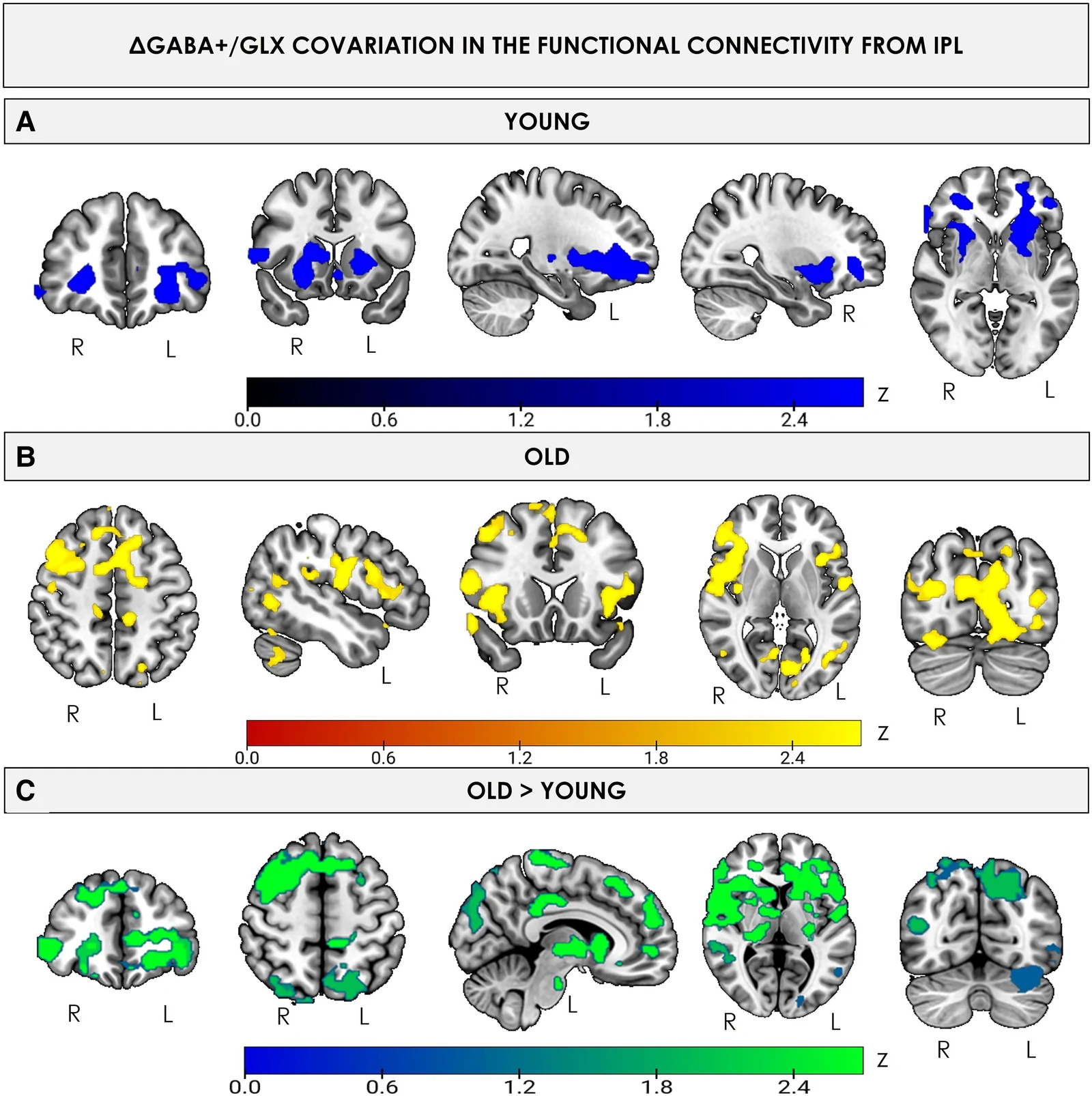
**ΔGABA+/Glx covariation effect in functional connectivity from the IPL.** (**A**) An increase in inhibition/excitation balance in the IPL during cognitive flexibility was associated with a negative coupling from this region in young adults (*n* = 34). (**B**) An increase in inhibition/excitation balance in the IPL during cognitive flexibility was associated with an enhanced functional connectivity from this region in older adults (*n* = 39). (**C**) Older adults showed higher functional connectivity from the IPL associated with an increase in inhibition/excitation in this region (*n* = 73). All maps: Psychophysiological interaction—covariation effect, z = 2.7, cluster correction *P* = 0.05.

The analogous analyses with the seed in the IPL revealed that in younger adults, a task-induced increase in the inhibitory tone in the IPL (ΔGABA+/Glx) related to a negative coupling between the IPL seed and the bilateral putamen and frontal pole, with the left insular cortex and with the inferior frontal gyrus (pars opercularis and pars triangularis), precentral gyrus, and caudate in the right hemisphere (Supplementary Table 5; Fig. 6). As ΔGABA+/Glx in IPL had been shown to negatively relate to cognitive flexibility performance in young adults,^9^ we extracted the beta parameters from the GLM of this neural connectivity map and assessed their correlation with behaviour. There was a positive correlation of the parameters of this map with behaviour (r = 0.37; *P* = 0.04; Supplementary Fig. 3).

In older adults a higher ΔGABA+/Glx (increase) was related to stronger connectivity of the IPL with the bilateral occipital fusiform gyrus, the central opercular cortex, the precentral gyrus, the postcentral gyrus, the left intracalcarine cortex and with the paracingulate gyrus, frontal orbital cortex and inferior frontal gyrus (pars opercularis) on the right hemisphere (Supplementary Table 5; Fig. 6). There was no significant correlation between the connectivity map parameters and behaviour (r = 0.01, *P* = 0.99).

When statistically comparing the association of ΔGABA+/Glx on functional connectivity between young and older individuals, the older individual showed an enhanced effect on the connectivity of the IPL seed with the superior temporal gyrus, parietal operculum cortex, middle frontal gyrus, and occipital pole in the right hemisphere, with the superior parietal lobule, occipital fusiform gyrus, cerebellum, brainstem, middle temporal gyrus and precuneus in the left hemisphere, and bilaterally with the lateral occipital cortex (Supplementary Table 5; Fig. 6). No significant differences were found in the Young > Old comparison.

Supplementary analyses showed that young individuals had higher levels of GABA and Glx in both voxels (GABA: Age: F_(1,76)_ = 22.33, *P* < 0.001, ղ^2^ = 0.12; Age X Voxel: F_(1,76)_ = 0.22, *P* = 0.64, ղ^2^ = 0.001; Glx: Age: F_(1,77)_ = 15.81, *P* < 0.001, ղ^2^ = 0.11; Age X Voxel: F_(1,77)_ = 0.07, *P* = 0.79, ղ^2^ < 0.001; Supplementary Table 6). Therefore, additional analyses were conducted to investigate the effect of the GABA and Glx baseline levels, separately, and their modulation, on the IFC and IPL connectivity. The results of these analyses are presented in the Supplementary material (Supplementary Tables 7–14, Supplementary Figs 4–11).

### Mediation of functional connectivity in the relationship between IPL ΔGABA+/Glx and behaviour

As the inhibitory/excitatory balance was a significant correlate of the functional connectivity of the IPL (previous section), and it is also related to behavioural performance,^9^ a mediation model was executed to investigate whether this relationship was mediated by the whole-brain functional connectivity from IPL. These analyses showed a null mediation effect of functional connectivity in young adults (Supplementary Table 15). In older adults, there was a significant mediation effect, but path b only showed a trend towards significance (β_b_ = −0.38, *P* = 0.06), not meeting the strict requirements for the mediation analysis (Supplementary Table 15).

**Figure 7:**
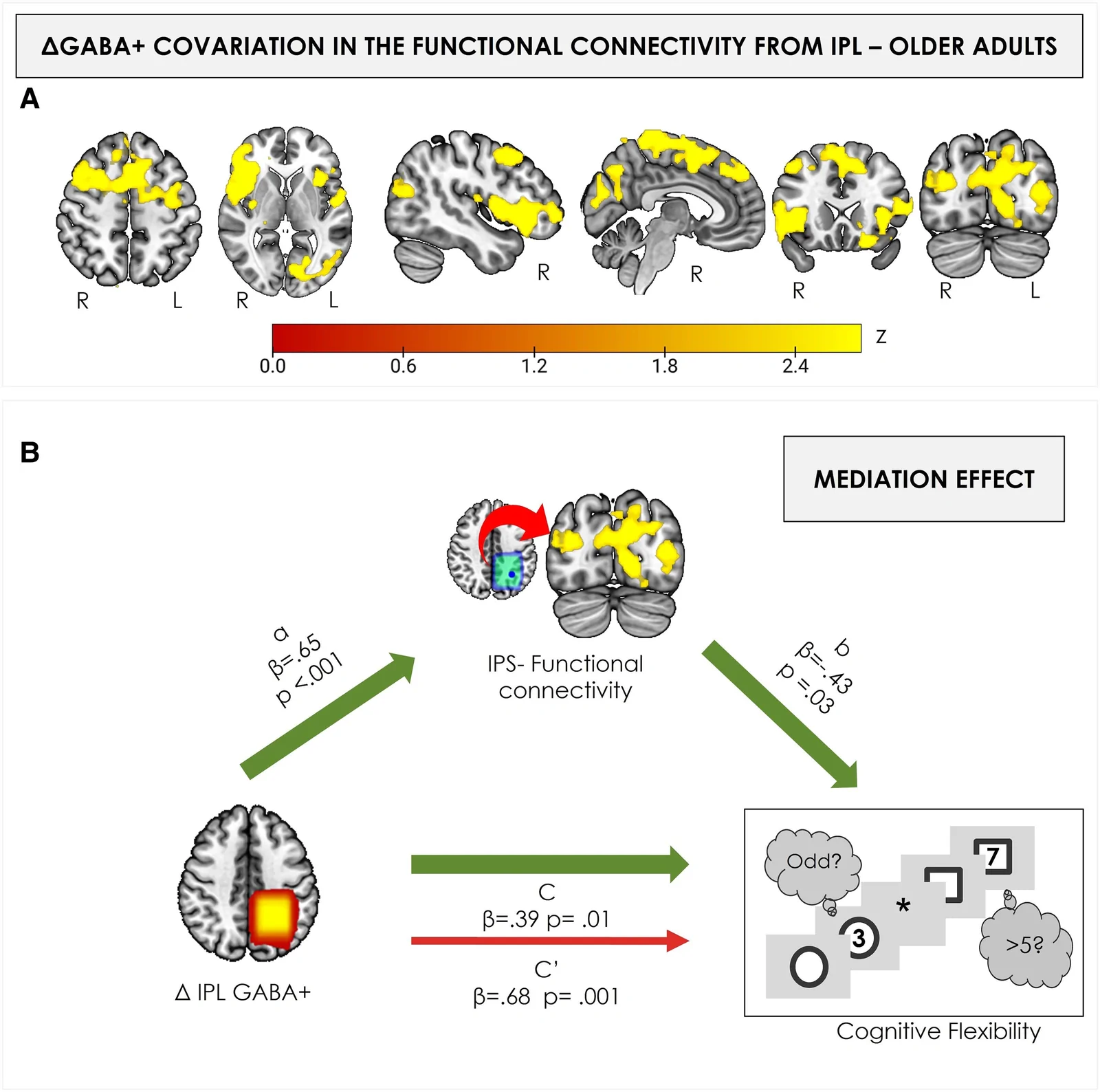
**ΔGABA+ covariation in functional connectivity from IPL and mediation analysis in older adults.** (A) An increase in the inhibition balance in the IPL during cognitive flexibility is associated with an increased functional connectivity from this region to the rest of the brain in older adults (Psychophysiological interaction—covariation effect, z = 2.7, cluster correction *P* = 0.05, *n* = 39). (**B**) The IPL functional connectivity associated with changes in GABA+ (ΔGABA+) in the IPL had an inconsistent mediation effect (see text) in the relationship between ΔGABA+ in the IPL and cognitive performance (Mediation analysis; *n* = 39).

As the path b was close to significance and as it had been reported that the relationship between ΔGABA+/Glx in IPL and cognitive flexibility was led by the modulation in GABA in older adults, indicating that an increase in GABA+ is related to better cognitive flexibility performance,^9^ we conducted this analysis with the parameters from the map of (whole-brain) functional connectivity modulated by GABA+ (that map indicated an enhanced IPL connectivity associated to an increase in GABA+; Fig. 7A), where there was a significant mediation effect in older adults (Supplementary Table 15, Fig. 7B; β_a_ = 0.65, *P* = 0.001; β_b_ = −0.43, *P* = 0.03; β_c’_ = 0.68, *P* = 0.001; β_ab_ = −0.28, CI: [−0.54, −0.08]). Note that in both analyses, the sign of path b was negative, with path a and path c being positive, indicating an inconsistent mediation effect. This pattern shows that whereas GABA+ modulation positively predicts IPL functional connectivity and behavioural performance, IPL functional connectivity negatively predicts behavioural performance (Supplementary Fig. 12).

Finally, we conducted more fine-grained analyses, choosing the connectivity between the IPL and the inferior frontal gyrus, putamen, and precuneus as mediators, which have been identified as core processing regions of cognitive flexibility.^2,30^ The results of these mediation analyses are presented in Table 1. These analyses showed that left IPL–precuneus connectivity had a significant, but negative, mediating effect on the relationship between ΔGABA/Glx in the IPL and cognitive flexibility performance in older adults (Supplementary Fig. 13). Finally, we aimed to investigate whether IPL functional connectivity would also be a significant mediator of the relationship between GABA+ modulation and cognitive flexibility. The results remained significant only for the negative mediation of the IPL-left Putamen connectivity (Table 1; Supplementary Fig. 14). For comparative purposes, the same analyses for young adults are also presented. Although none of these mediations was significant, the results showed that stronger connectivity between the left IPL and bilateral putamen was associated with better cognitive flexibility performance in young adults (Supplementary Fig. 15). For complementary purposes, in Supplementary Figs 13–15 we include correlation plots from the mediation analyses where path *a* and path *b* were significant.

**Table 1:** Mediation analyses to assess the mediating role of functional connectivity (IPL seed; whole brain and region-specific) in the relationship between metabolic modulation and behaviour

Mediation of IPL-brain region connectivity								
YOUNG	βc	βc’	βab	BootSE	BootLLCI	BootULCI	βa	βb
ΔGABA	*P*	*P*	std				*P*	*P*
IPL-Precuneus	−0.27	−0.23	−0.03	0.06	−0.14	0.07	−0.18	0.19
	0.14	0.21					0.34	0.28
IPL-Left Putamen	−0.27	−0.05	−0.19	0.14	−0.55	0.01	−0.46	0.41
	0.14	0.78					0.01	0.05
IPL-Right Putamen	−0.27	−0.05	−0.20	0.14	−0.53	0.01	−0.39	0.51
	0.14	0.81					0.03	0.01

OLD	βc	βc’	βab	BootSE	BootLLCI	BootULCI	βa	βb
ΔGABA+	*P*	*P*	std				*P*	*P*
IPL-Precuneus	0.39	0.54	−0.15	0.08	−0.32	0.003	0.44	−0.33
	0.01	0.002					0.005	0.04
IPL-Left Putamen	0.39	0.53	−0.14	0.07	−0.28	−0.007	0.41	−0.33
	0.01	0.002					0.009	0.05
IPL-Right Putamen	0.39	0.51	−0.11	0.06	−0.25	0.001	0.33	−0.31
	0.01	0.002					0.04	0.05

Fx conn—functional connectivity, pt—pars triangularis, po—pars opercularis.

## Discussion

We investigated functional connectivity patterns from two important executive function hubs, the IFC and IPL, during cognitive flexibility performance in young and older adults. We observed a significant functional connectivity pattern from the IPL in older individuals, showing functional coupling with the cerebellum, precuneus, and hippocampus during switching trials. Further, we examined whether modulations in the inhibition/excitation balance resulting from cognitive flexibility performance were related to functional connectivity. We observed that the inhibitory-excitatory balance in IFC and IPL significantly affected the functional connectivity of these regions during cognitive flexibility in young and older adults. The functional connectivity pattern associated with GABA+/Glx modulation in older adults showed an inconsistent mediation effect on the relationship between inhibitory/excitatory tone modulation and cognitive flexibility performance. Further analyses showed that the functional connectivity between the IPL and the precuneus in older adults was a significant (but inconsistent) mediator of the relationship between GABA+/Glx modulation and cognitive flexibility performance. Likewise, the functional connectivity between the IPL and the left putamen mediated the relationship between GABA+ modulation and behavioural performance. No mediation effect was observed in young adults. Finally, the connectivity between IPL and bilateral putamen was related to cognitive flexibility performance in both groups, but in opposite directions.

### Behaviour and fMRI responses

Young individuals performed better during the fMRI session than older individuals. However, both samples performed similarly during the last training block. Furthermore, our observations regarding the MRI BOLD activation pattern are consistent with previous reports, showing activation in the frontoparietal network.^2,31^

### Functional connectivity from IFC and IPL

When investigating functional connectivity from the IFC and IPL during flexible behaviour, the only significant pattern was from the IPL in older adults, in which IPL activation during cognitive flexibility was coupled with that from the precuneus, the fusiform cortex (extending to the hippocampus), and the cerebellum. Structural and effective connectivity between the IPL and the precuneus has been reported,^32,33^ and it has been proposed that the precuneus is involved in flexibility and executive functions via the IPL.^34^ Being richly connected, the precuneus has a high centrality in the cortical network^35^ and is involved in different higher-order processes such as the encoding of verbal abstract rules.^36,37^ On the other hand, the cerebellum is involved in working memory,^2^ and the hippocampus plays a key role in memory encoding.^38^ Therefore, the simultaneous coupling of IPL with precuneus, cerebellum, and hippocampus may imply the encoding of abstract rules that occurs during the early phase of the task (i.e. learning) in older adults. Whereas young adults also require the encoding of abstract rules, the fact that older adults took longer to learn the task and that they exhibited a stronger and more distributed BOLD response during cognitive flexibility may have contributed to the observation of this connectivity pattern only in older adults, which could indicate a higher demand for resources to perform the task.

This connectivity pattern was not related to behaviour, suggesting that this network is relevant to the minimum cognitive requirements for task performance (presumably the encoding of abstract rules). The lack of significant functional connectivity patterns from the IFC in both samples may suggest heterogeneous connectivity across participants and may relate to different strategies during task performance. In addition, it is worth mentioning that PPI has low statistical power, especially in event-related protocols.^24^

### Functional connectivity associated with inhibition/excitation modulation

The modulation in the inhibitory/excitatory tone had differential effects on functional connectivity across regions and age groups. The opposite effect on functional connectivity (positive or negative coupling) across regions could possibly be due to distinct cytoarchitecture and physiology within each region,^39^ which implies that different regions have distinct functions and demands, and differential use of neurometabolites such as GABA and glutamate.

The significant differences between young and older adults highlight that ageing is accompanied by important physiological changes underlying flexible behaviour, and specifically that neurochemical dynamics have a distinct effect on the brain functional networks. Such differences may reflect adaptive processes during ageing, possibly driven by other neurophysiological and structural changes such as reduction in grey matter,^40,41^ changes in functional and structural connectivity,^17,42^ as well as neurophysiological changes, such as reductions in the basal level of GABA+ and Glx and changes in neurometabolic dynamics.^9,43,44^

Whereas a higher inhibitory tone has been associated with reduced functional connectivity during resting state,^10,45-47^ the current results showed that an increase in local inhibitory tone can also be associated with the strengthening of particular functional networks during cognitive flexibility task performance.

An increase in the inhibitory tone (GABA+/Glx) in the IFC in young adults was associated with increased connectivity of this region with the superior frontal gyrus (involved in executive control) and with the precentral gyrus and the caudate (both related to movement). This indicates that an increase in GABA+/Glx in the IFC may facilitate the coordination of an executive motor control network (necessary for button pressing according to specific rules). In contrast, in older adults, an increase in the inhibitory tone was related to a negative coupling between the IFC and the caudate, the insula, the inferior frontal gyrus, and the operculum in the right hemisphere. This negative coupling indicates that when the IFC is more active, those structures become less active, and vice versa. Furthermore, in older adults, there is an increase in the bilateralization of cognitive functions, known as the HAROLD effect^48^ (Supplementary Fig. 2). An increase in inhibitory tone in the IFC seems to suppress that effect (in the IFG and adjacent regions) during cognitive flexibility in older adults.

An increase in the inhibitory tone in the IPL was associated with a negative functional coupling with regions associated with flexible behaviour (the bilateral putamen, the right caudate),^1,2^ motor control (the right precentral gyrus)^49^ and action monitoring (bilateral frontal pole)^50^ in young individuals. As young individuals who had a reduced inhibitory tone showed higher flexibility,^9^ a reduced inhibitory tone could be activating this network while facilitating switching behaviour. In this regard, although there was a positive correlation between functional connectivity and behaviour, this association was no longer significant in the regression model after including neurometabolic modulation (as observed in mediation analyses). This indicates that functional connectivity relates to behaviour only in the degree that it is related to (possibly modulated by) neurometabolic modulation. In addition, the IPL-putamen connectivity positively predicted cognitive flexibility performance in young adults after controlling for neurometabolic modulation (showing its relevance in cognitive flexibility performance independent of neurometabolic modulation).

In older adults, an increase in the GABA+/Glx balance in the IPL was associated with stronger connectivity with visual (occipital cortex), motor (precentral gyrus), and somatosensory (postcentral cortex and operculum) processing regions. However, there was no direct significant association between the aforementioned connectivity pattern and behaviour, and this connectivity pattern was not a mediator in the relationship between functional connectivity and behaviour.

Further analyses indicated that an increase in GABA+ (also related to better performance in older adults)^9^ was related to an enhanced connectivity of IPL with prefrontal regions (orbital, inferior frontal and superior frontal cortices, frontal pole and supplementary motor cortex) and visual (lateral occipital, intracalcarine and cuneal cortices) and motor processing regions (precentral gyrus and supplementary motor cortex area). The abovementioned prefrontal regions are relevant in executive control.^3,51-54^ In older adults, increased inhibitory tone in the IPL seemed to facilitate selective (executive) functional connectivity. The activation of this network being associated with an upregulation of GABA+ only in older adults could be related to a higher cognitive demand in this sample as compared to the young sample.

We anticipated that a functional connectivity pattern associated with increased inhibition would relate to better cognitive performance, based on the reported association between increased inhibitory tone in the IPL and better cognitive flexibility in older adults.^9^ Mediation analyses showed that this connectivity pattern mediated the relation between neurometabolic modulation and behaviour, but the effect was negative: whereas an increase in GABA+ predicted better performance and stronger connectivity, stronger connectivity predicted lower flexible performance. This also implied that, after controlling for the effect of functional connectivity, the relationship between GABA+ modulation and behaviour became stronger. This paradox could possibly be explained by the fact that an increase in GABA+ may actually facilitate a connectivity pattern that supports flexible behaviour. However, the relationship between enhanced connectivity and worse behaviour in older adults (controlling for neurometabolic modulation) may be related to a reduced capacity to suppress irrelevant network communication.^55^

The same argument may apply for the left IPL–precuneus (mediating the GABA+/Glx modulation-behaviour relationship) and left IPL–left putamen (mediating the GABA+ modulation-behaviour relationship) functional connectivity. These connectivity patterns seemed to be facilitated by increased inhibitory tone in older adults, but, at the same time, predicted poorer performance. In young adults, functional connectivity did not mediate the relationship between neurometabolic modulation and behaviour. However, stronger left IPL–bilateral putamen connectivity was associated with better performance in this sample. Interestingly, in older adults, stronger left IPL–bilateral putamen and left IPL–precuneus connectivity were associated with poorer cognitive flexibility performance. Whereas fronto-subcortical circuits have been widely investigated in the context of cognitive performance and flexibility,^56-58^ the current evidence suggests that parietal-putamen connectivity also plays a role in cognitive flexibility.

Finally, this study focused on the association between inhibition/excitation modulation and functional connectivity, but our supplementary analyses showed that baseline levels of GABA+ and Glx also play distinct roles in the functional connectivity of the IFC and IPL, with important age differences. Furthermore, these supplementary analyses showed that the inhibition/excitation relationship with functional connectivity is mostly, but not exclusively, led by GABA+.

### Limitations

One limitation of this study is that no directional conclusions can be drawn. Therefore, it is not possible to know whether metabolic changes drive functional connectivity patterns or whether these are intertwined processes that affect each other. Future studies using non-invasive brain stimulation techniques or pharmacological interventions may help shed light on causal mechanisms. A second limitation is that the functional connectivity and the metabolites were measured at different time points. Future studies using simultaneous acquisition of fMRI and MRS could perhaps overcome this limitation.^6,8,59^

Finally, this study was conducted with participants recruited in Belgium, with a big proportion of them having higher education studies (both samples) and who voluntarily registered for the study. Due to the lack of literature on the effect of sociodemographic variables on neurometabolic dynamics, we cannot estimate the generalizability of the current findings.

## Conclusion

In summary, this study sheds light on the regulation of the inhibition/excitation balance and functional connectivity during cognitive flexibility in the IPL, and on their association with behavioural performance. It also revealed important age-related differences in the metabolic dynamics and associated functional connectivity during flexible behaviour. In particular, young adults showed stronger functional connectivity associated with increased inhibitory tone in the IFC than older adults, but this pattern was reversed for the IPL. In addition, whereas in young adults, the connectivity between the left IPL and the bilateral putamen predicted better cognitive flexibility, this relationship had an opposite direction in older adults. In sum, these results show that neurometabolic dynamics and functional connectivity underlying cognitive flexibility undergo substantial changes during ageing.

## Supplementary Material

fcag319_Supplementary_Data

## Data Availability

The dataset used for the present study can be found in the OSF repository: https://osf.io/t8dzn/overview?view_only=9cf0a6744ed04ca597146fa730bc37c4
