# Supplementary material for: Inhibition/excitation balance is associated with functional connectivity during cognitive flexibility performance
Source: Brain Commun. 2026 Sep 28;8(5):fcag319. doi: 10.1093/braincomms/fcag319 (PMC13616520; doi:10.1093/braincomms/fcag319)
Supplement: Supplementary file 1 [file fcag319_supplementary_data.pdf]

Supplementary Table 1. MRSinMRS checklist. [1]

**1. Hardware**	
a. Field strength	3T
b. Manufacturer	Philips
c. Model (software version)	Achieva
d. RF coils	^1H 32-channel phased-array head coil for receive; body coil for transmit
e. Additional hardware	n/a
**2. Acquisition**	
a. Pulse sequence	HERMES
b. Volume of interest (VOI) locations	Inferior frontal cortex (IFC); inferior parietal lobe (IPL)
c. Nominal VOI sizes	IFC: $4 \times 2.5 \times 2.5$ cm^3; IPL: $3 \times 3 \times 3$ cm^3
d. Repetition time (TR), echo time (TE)	TR/TE = 2000/80 ms
e. Total number of excitations or acquisitions per spectrum	320 water-suppressed; 16 unsuppressed
f. Additional sequence parameters	Spectral width: 5000 Hz<br>Spectral points: 4096
g. Water suppression method	VAPOR
h. Shimming method, reference peak, and thresholds for "acceptance of shim" chosen	PB-auto (pencil-beam projection)
i. Triggering or motion correction method	n/a
**3. Data analysis methods and outputs**	
a. Analysis software	Gannet (v3.3.0)
b. Processing steps deviating from quoted reference or product	Standard Gannet pipeline[2]
c. Output measure<br>Processing steps deviating from quoted reference or product	GABA+/Glx (each metabolite reference to water)

d. Quantification references and assumptions, fitting model assumptions	The tissue fractions were used to correct the obtained GABA+ for partial volume effects (Harris et al., 2015)[2].
**4. Data Quality**	
a. Reported variables (SNR, Linewidth (with reference peaks))	
b. Data exclusion criteria	GABA+ estimates with a fit error greater than 3 std. from the group mean were excluded.
c. Quality measures of postprocessing model fitting	Fit error.
d. Sample spectrum	Provided in the main text.

[1] Minimal reporting standards as recommended in: Lin A, Andronesi O, Bogner W, et al. Minimum Reporting Standards for in vivo Magnetic Resonance Spectroscopy (MRSinMRS): Experts' consensus recommendations. *NMR Biomed*. May 2021;34(5):e4484. doi:10.1002/nbm.4484

[2] Harris AD, Puts NA, Edden RA. Tissue correction for GABA-edited MRS: Considerations of voxel composition, tissue segmentation, and tissue relaxations. *Journal of Magnetic Resonance Imaging.* 2015;42(5): 1431-1440.

Supplementary Figure 1. Behavioral performance in the cognitive flexibility task in young (red) and older (blue) adults (Young n=34, Old n=39). Young adults showed a better performance than older adults during the task (t-test: $t_{(70)}$= -3.33, p =<.001, *d*=0.79; Cognitive flexibility index (Repeat hits minus switch hits) young: mean= -7.17, SD= 8.61, old: mean=-14.31, SD= 9.22).

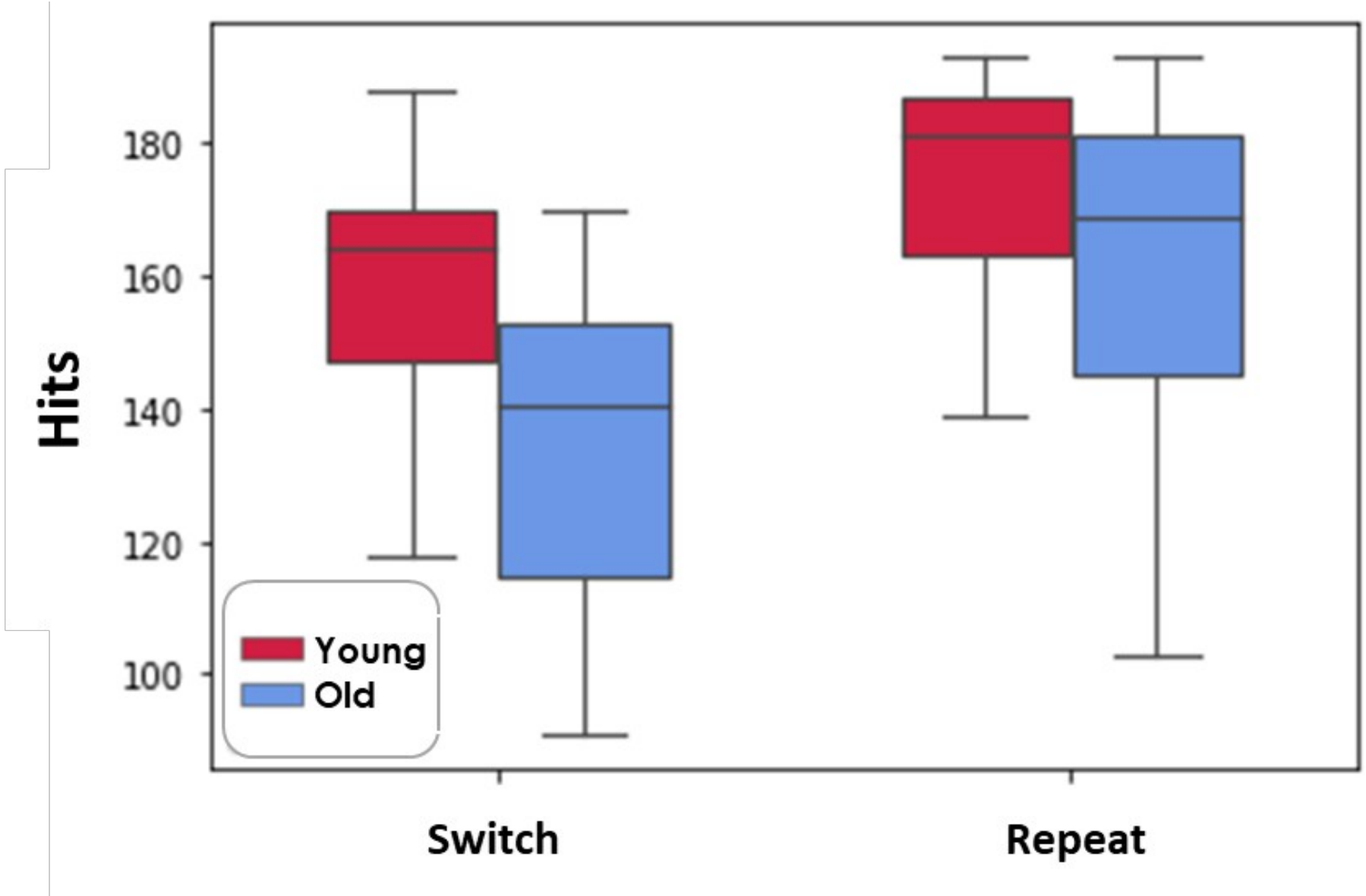

Supplementary Figure 2. Group-average BOLD activity in young (top) and older (bottom) individuals during the performance of the cognitive flexibility task (Switch > Repeat (General Linear Model); $z < 2.7$, cluster level threshold-corrected at $p < 0.05$). The bottom panel shows significant differences according to the age group (Old >Young; no significant differences were observed in the Young>Old contrast). The bottom numbers indicate the z-coordinate relative to the MNI152 template. L, left hemisphere. (Young n=34, Old n=39).

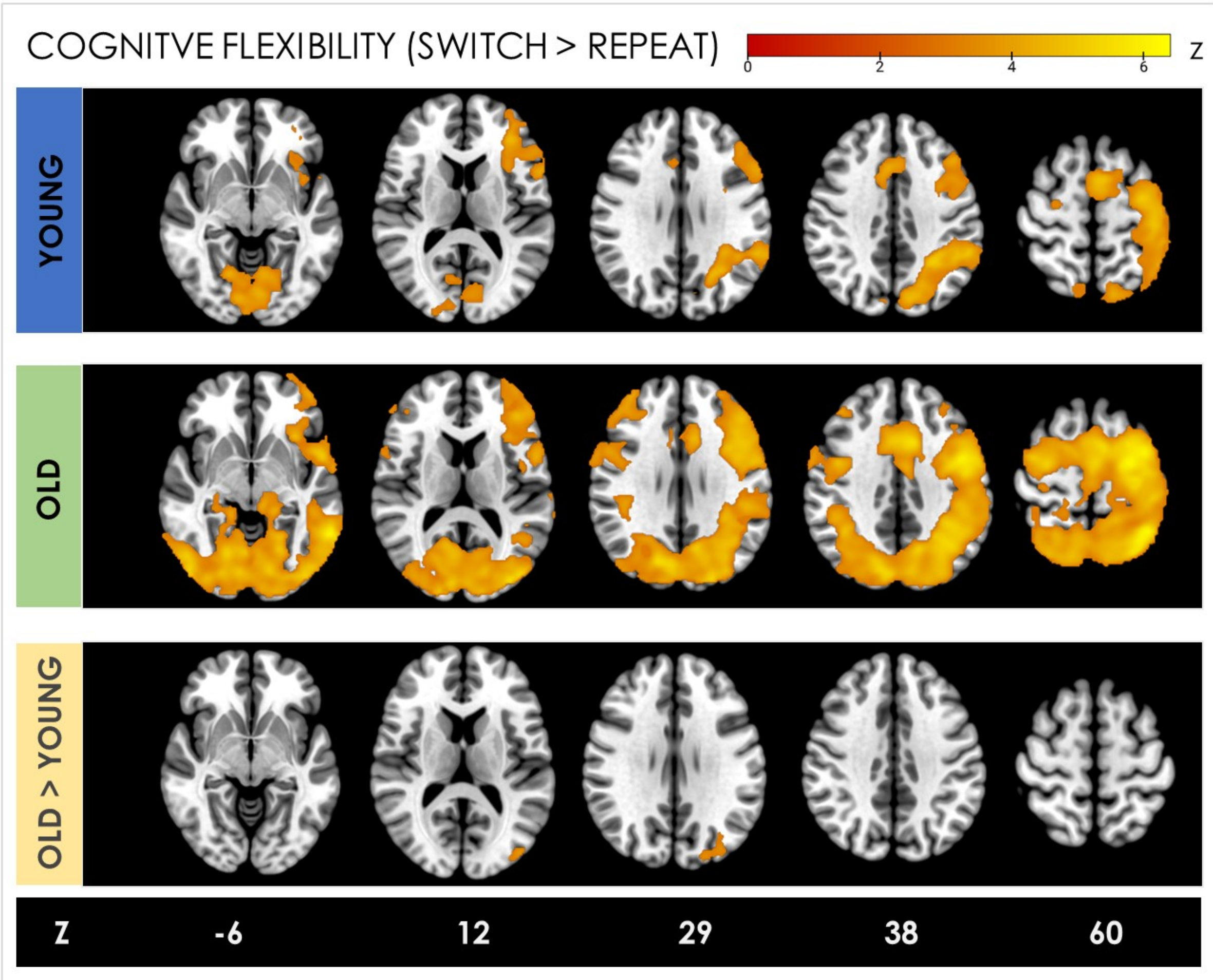

Supplementary Table 2. Local maxima of the average BOLD activity in young and older participants and the difference (Switch > Repeat)

	Z (local max)	x	y	z
**Young**				
Precuneus/Superior parietal lobe	5.49	-8	-80	48
Juxtapositional Lobule cortex/ Premotor cortex	5.45	-6	6	58
Superior parietal lobule / Intraparietal sulcus	5.37	-32	-54	42
Angular gyrus/Inferior parietal lobule	4.99	-48	-52	58
Intracalcarine cortex/Visual cortex	4.68	-8	-84	2
**Old**				
Premotor cortex/Precentral gyrus	-28	-10	64	6.7
Superior parietal lobe/precuneus	-12	-66	48	6.28
Anterior intraparietal sulcus / Angular gyrus	-40	-56	60	6.19
Superior parietal lobe	-28	-60	48	6.05
Inferior parietal lobule	-32	-70	56	6.04
Insular cortex	36	14	4	4.57
Callosal body	26	36	0	3.9
Frontal orbital cortex	34	30	2	3.85
Insular cortex	46	6	-2	3.45
**Young > Old BOLD activity (Switch > Repeat)**				
None	--	--	--	--
**Old > Young BOLD activity (Switch > Repeat)**				
Lateral occipital cortex/Inferior parietal lobule	4.3	-40	-88	20
Lateral occipital cortex/Visual cortex	3.51	-36	-76	6
Lateral occipital cortex/Superior parietal lobule	3.65	22	-76	48
Lateral occipital cortex	3.73	-58	-72	-4
Inferior temporal gyrus	4.05	62	-60	-16
Lateral occipital cortex	3.72	54	-66	-6
Middle Temporal gyrus	3.58	64	-62	-2

Supplementary Table 3. Functional connectivity from IPL

**Area**	**Z (local max)**	**x**	**y**	**z**
**Young**				
--	--	--	--	--
**Old**				
Cerebellum V	4.13	26	-36	-30
Cerebellum VI	3.66	34	-38	-16
Cerebellum VI	3.65	10	-68	-16
Lingual Gyrus	3.46	-10	-86	-10
Occipital Fusiform Gyrus	3.42	-20	-86	-12
Temporal Occipital Fusiform Cortex	3.42	-26	-46	-12
Left Cerebral White Matter	4.36	-16	-48	40
Superior Parietal Lobule	3.66	-26	-52	40
Postcentral Gyrus	3.63	-58	-10	30
Left Cerebral White Matter	3.55	-36	-16	30
Postcentral Gyrus	3.32	-42	-26	36
Precuneus	3.1	0	-46	48
Planum Temporale	3.41	-62	-30	14
Planum Temporale	3.31	-62	-36	14
Superior Temporal Gyrus	3.21	-68	-24	10
Middle Temporal Gyrus	3.2	-70	-34	-4
Middle Temporal Gyrus	3.03	-66	-6	-14
**Young > Old**				
None	--	--	--	--
**Old > young**				
Occipital Fusiform Gyrus	3.67	26	-86	-12
Occipital Fusiform Gyrus	3.43	38	-68	-12
Cerebellum	3.38	44	-68	-18
Cerebellum	3.32	26	-82	-22
Occipital Pole	3.23	14	-92	12

Supplementary Table 4. Association between ΔGABA+/Glx and functional connectivity in the IFC.

Area	Z (local max)	x	y	z
**Young**				
Superior Frontal Gyrus	5.04	-4	30	60
Superior Frontal Gyrus	4.79	-2	20	58
Superior Frontal Gyrus	4.78	-4	24	56
Superior Frontal Gyrus	4.16	18	26	56
Superior Frontal Gyrus	4.02	6	26	56
Precentral Gyrus	4.36	38	-2	64
Caudate	3.67	20	22	6
Caudate	3.38	8	12	-2
**Old**				
Caudate	4.24	20	12	16
Insular cortex	3.69	34	2	16
Insular cortex	3.3	38	10	6
Inferior Frontal Gyrus pars opercularis	3.3	50	10	14
Inferior Frontal Gyrus pars triangularis	3.05	48	24	6
Central Opercular Cortex	2.98	52	-4	16
**Young > Old**				
Central Opercular Cortex	4.01	40	2	12
Precentral Gyrus	3.87	54	8	8
Precentral Gyrus	3.76	-2	-16	70
Precentral Gyrus	3.66	-18	-16	66
Frontal Pole	3.87	40	42	0
Superior Frontal Gyrus	4.53	-2	20	58
Superior Frontal Gyrus	4.46	-4	24	56
Superior Frontal Gyrus	3.89	18	28	54
Juxtapositional Lobule Cortex / Supplementary Motor Cortex	4.12	2	8	62

**Old > Young**				
---	--	--	--	--

Supplementary Table 5. Positive effect of ΔGABA+/Glx on the IPL functional connectivity.

**Young**	**Z (local max)**	x	y	z
Putamen	4.39	-22	14	-2
Putamen	3.96	22	16	-6
Putamen	3.94	20	16	-10
Putamen	3.77	24	20	2
Putamen	2.47	30	-8	-4
Frontal Pole	4.25	-28	44	-8
Frontal Pole	3.61	-22	54	-4
Frontal Pole	3.73	34	46	-4
Precentral Gyrus	3.88	64	10	6
Insular Cortex	3.5	-30	22	0
Inferior Frontal Gyrus pars opercularis	3.95	62	14	4
Inferior Frontal Gyrus pars triangularis	3.64	60	24	2
Inferior Frontal Gyrus pars triangularis	3.47	58	32	0
Caudate	3.97	16	12	10
**Old**				
Intracalcarine cortex	3.83	-12	-78	2
Intracalcarine cortex	3.52	-22	-68	4
Occipital Fusiform Gyrus	3.78	34	-76	-20
Occipital Fusiform Gyrus	3.72	-20	-84	-4
Occipital Fusiform Gyrus	3.67	-16	-76	-6
Central Opercular Cortex	3.7	54	-72	-18
Precentral Gyrus	3.88	4	-28	78
Precentral Gyrus	3.62	2	-18	68
Precentral Gyrus	3.78	-54	-4	16
Precentral Gyrus	3.71	-54	-6	20
Precentral Gyrus	3.41	14	-22	44

Postcentral Gyrus	3.87	12	-38	66
Postcentral Gyrus	3.83	6	-32	78
Postcentral Gyrus	3.8	-52	-18	30
Postcentral Gyrus	3.74	-58	-10	26
Paracingulate Gyrus	3.76	-4	10	52
Superior Frontal Gyrus	3.64	12	-6	66
Central Opercular Cortex	4.23	50	6	6
Central Opercular Cortex	3.69	58	-2	12
Central Opercular Cortex	3.66	-58	-4	4
Central Opercular Cortex	3.65	-48	-12	20
Frontal Orbital Cortex	4.1	34	28	0
Frontal Orbital Cortex	4.01	50	28	-10
Inferior Frontal Gyrus, pars opercularis	3.9	58	16	12
Frontal Operculum Cortex	3.62	38	20	6
Posterior Cingulate Gyrus	3.74	-6	-36	26
Posterior Cingulate Gyrus	3.37	10	-32	34
Posterior Cingulate Gyrus	3.13	8	-22	38
Posterior Cingulate Gyrus	3.08	8	-30	28
**Old > Young**				
Superior Temporal Gyrus	4.63	58	2	-14
Parietal Operculum Cortex	4.54	36	-24	18
Middle Frontal Gyrus	4.45	38	12	52
Superior Parietal Lobule	4.37	-32	-52	38
Occipital Fusiform Gyrus	4.86	-38	-66	-20
Cerebellum VI	3.89	-38	-46	-28
Cerbellum VI	3.86	-38	-46	-32
Brainstem	3.86	-4	-36	-34
Brainstem	3.43	-12	-30	-34
Middle Temporal Gyrus	3.8	-54	-62	2
Lateral Occipital Cortex	4.18	-22	-70	52
Lateral Occipital Cortex	4.03	14	-84	48
Lateral Occipital Cortex	3.55	-26	-66	40
Lateral Occipital Cortex	3.3	-24	-62	52
Occipital Pole	3.91	14	-92	32

Precuneus	3.6	-8	-74	46
**Young > Old**				
--	--	--	--	--

Supplementary Table 6. Baseline levels of GABA and Glx in young and older adults.

	YOUNG<br>Mean<br>SD	OLD<br>Mean<br>SD
GABA IFC<br>(baseline)	2.51<br>(.39)	2.25<br>(.44)
GABA IPL<br>(baseline)	2.27<br>(.26)	2.03<br>(.29)
Glx IFC<br>(baseline)	7.14<br>(.77)	6.61<br>(.73)
Glx IPL<br>(baseline)	6.76<br>(.69)	6.26<br>(.72)

Supplementary Figure 3. Pearson correlation between functional connectivity from IPL (correlated to ∆GABA/Glx) and cognitive flexibility performance in young adults (n=34). Each datapoint represents the data from one participant.

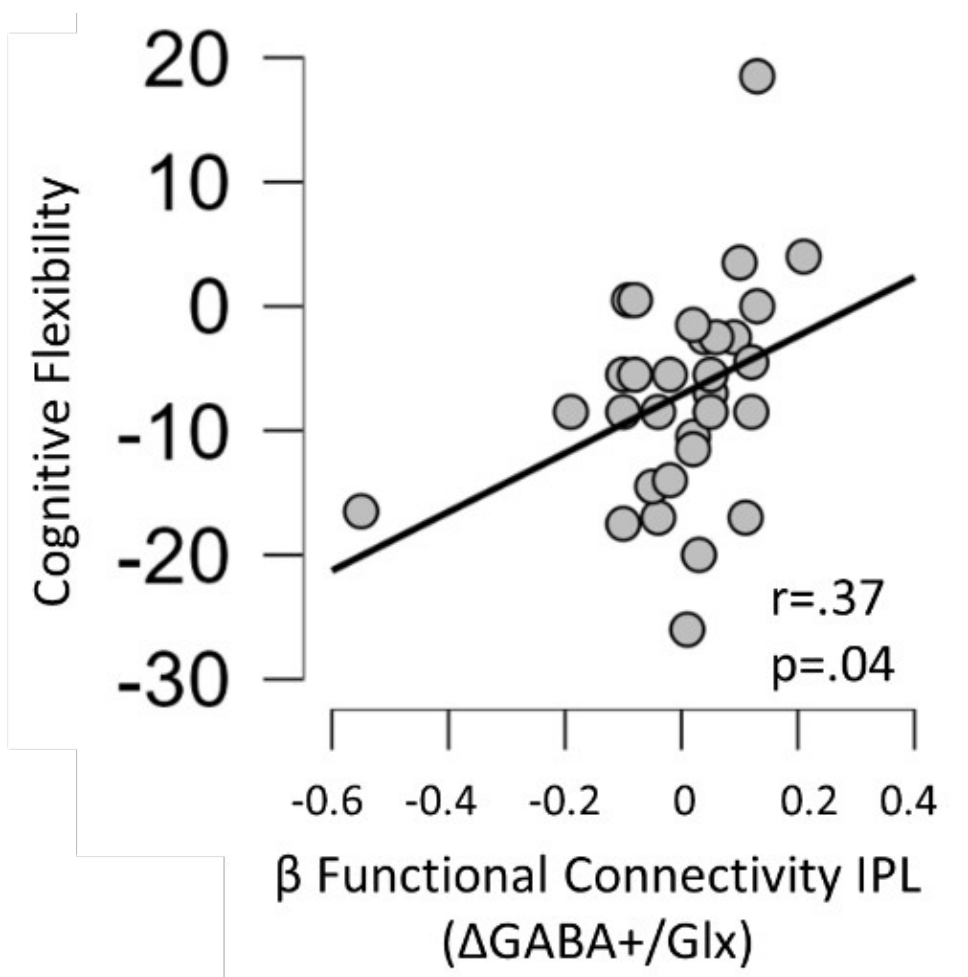

Supplementary Figure 4. Association between baseline GABA+ and functional connectivity in the IFC (Young n=34, Old n=37). In young adults higher levels of GABA+ were related to a weaker functional connectivity of IFC with the left precentral and superior frontal gyrus. In older adults, higher levels of GABA+ were related to a stronger functional connectivity of the IFC with the frontal pole, the post-central gyrus cerebellum and occipital regions. The relationship of baseline GABA+ on the functional connectivity of IFC was significantly different between young and older adults, with older adults having a stronger functional connectivity as baseline GABA+ levels are higher. All maps: Pychophysiological interaction – covariation effect, z=2.7, cluster correction p=.05.

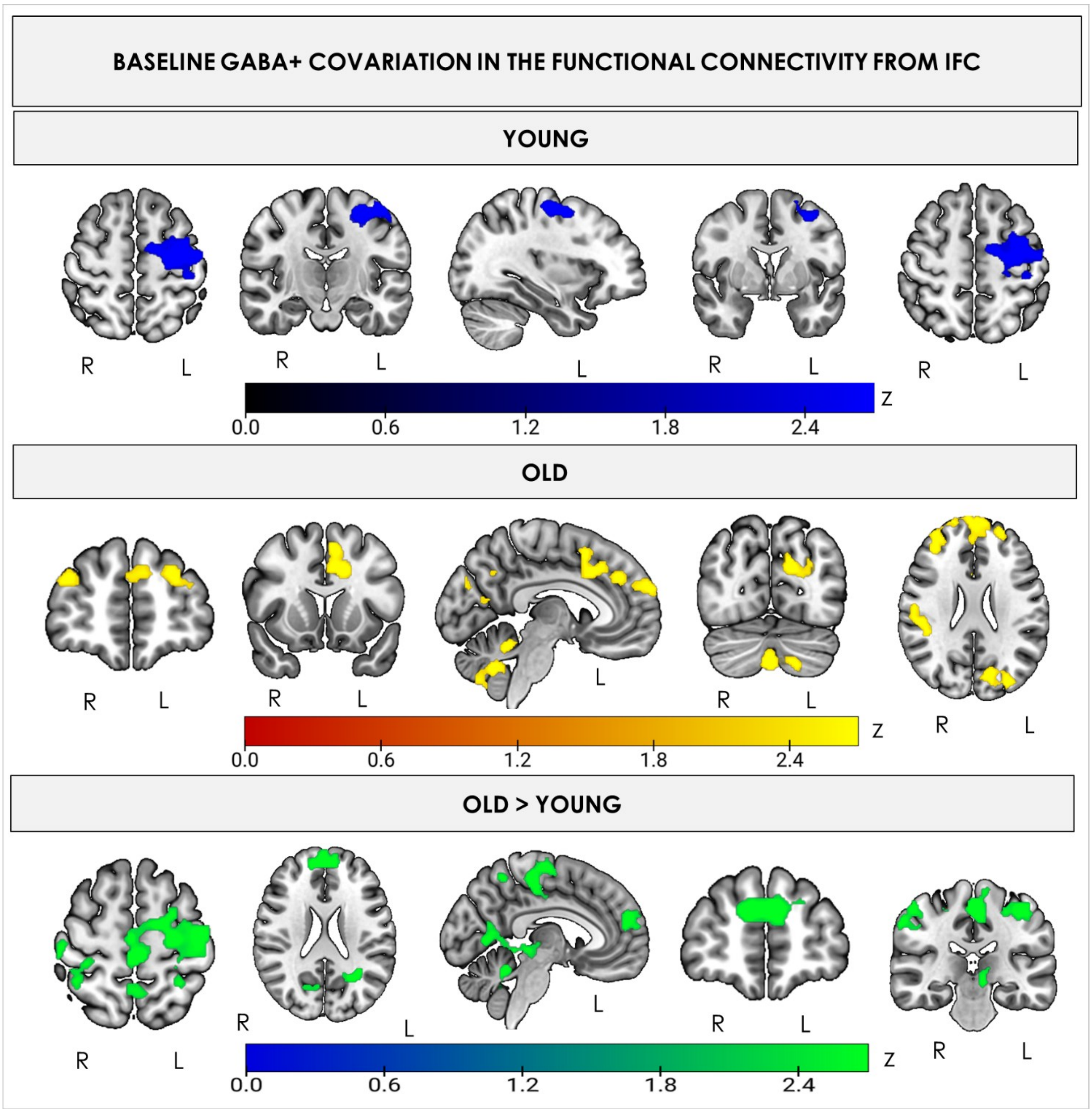

Supplementary Table 7. Association of baseline GABA+ in IFC with functional connectivity.

	Region	Z	x	y	z
Young (Negative Effect)					
	Precentral Gyrus	4.08	-30	-16	58
	Precentral Gyrus	3.82	-44	-14	62
	Precentral Gyrus	3.65	-36	-4	54
	Superior Frontal Gyrus	3.11	-16	-8	58
	Postcentral Gyrus	2.75	-40	-28	56
	Superior Frontal Gyrus	2.61	-24	-4	58
Old (Positive Effect)					
	Frontal Pole	3.66	-2	64	26
	Frontal Pole	3.37	36	54	24
	Frontal Pole	3.34	20	72	16
	Parietal operculum cortex	3.56	46	-32	30
	PostCentral Gyrus	3.48	64	-18	36
	PostCentral Gyrus	3.45	58	-14	32
	Parietal Operculum Cortex	3.34	56	-26	20
	Cerebellum	3.77	2	-60	-36
	Cerebellum	3.76	22	-60	-28
	Lateral Occipital Cortex	3.59	-34	-82	24
	Cuneal Cortex	3.39	-14	-76	26
	Lateral Occipital Cortex	3.13	-20	-84	20
	Lateral Occipital Cortex	3.11	-30	-84	32
	Precuneus	2.89	-12	-62	36
	Supracalcarine Cortex	2.8	-16	-68	18
Young > Old					
	--	--	--	--	--
Old > Young					
	Precentral Gyrus	4.11	-2	-26	54
	Precentral Gyrus	3.8	-28	-18	58
	Precentral Gyrus	3.75	-44	-16	62
	Postcentral Gyrus	3.7	-40	-28	54
	Precentral Gyrus	3.67	-36	-6	54
	Precentral Gyrus	3.63	-42	-12	56
	Supracalcarine Cortex	3.88	-14	-66	16
	Supramarginal Gyrus	3.87	-46	-44	10
	Lingual Gyrus	3.36	-2	-64	4
	Superior Parietal Lobule	3.64	36	-38	54
	PostCentral Gyrus	3.4	50	-22	56
	Supramarginal Gyrus	3.38	62	-26	44
	Precentral Gyrus	2.67	40	-12	52

	Superior Frontal Gyrus	3.49	10	54	20
	Frontla pole	3.43	2	58	24
	Superior Frontal Gyrus	3.41	10	52	24
	Superior Frontal Gyrus	2.95	-6	52	16
	Frontal Pole	2.88	-8	60	14
	Frontal Pole	2.85	-14	72	14
	Temporal Occipital Fusiform Cortex	3.5	40	-54	-14
	Intracalcarine Cortex	3.43	26	-68	10
	Intracalcarine Cortex	3.13	26	-60	-6
	Occipital Fusiform Gyrus	3.13	26	-72	-4
	Cuneal Cortex	2.94	18	-68	20
	Cuneal cortex	2.77	12	-74	0

Supplementary Figure 5. Null association between baseline Glx and functional connectivity in the IFC (Young n=34, Old n=37). The baseline levels of Glx did not hold any significant relation with the functional connectivity of IFC in any group (Pychophysiological interaction – covariation effect).

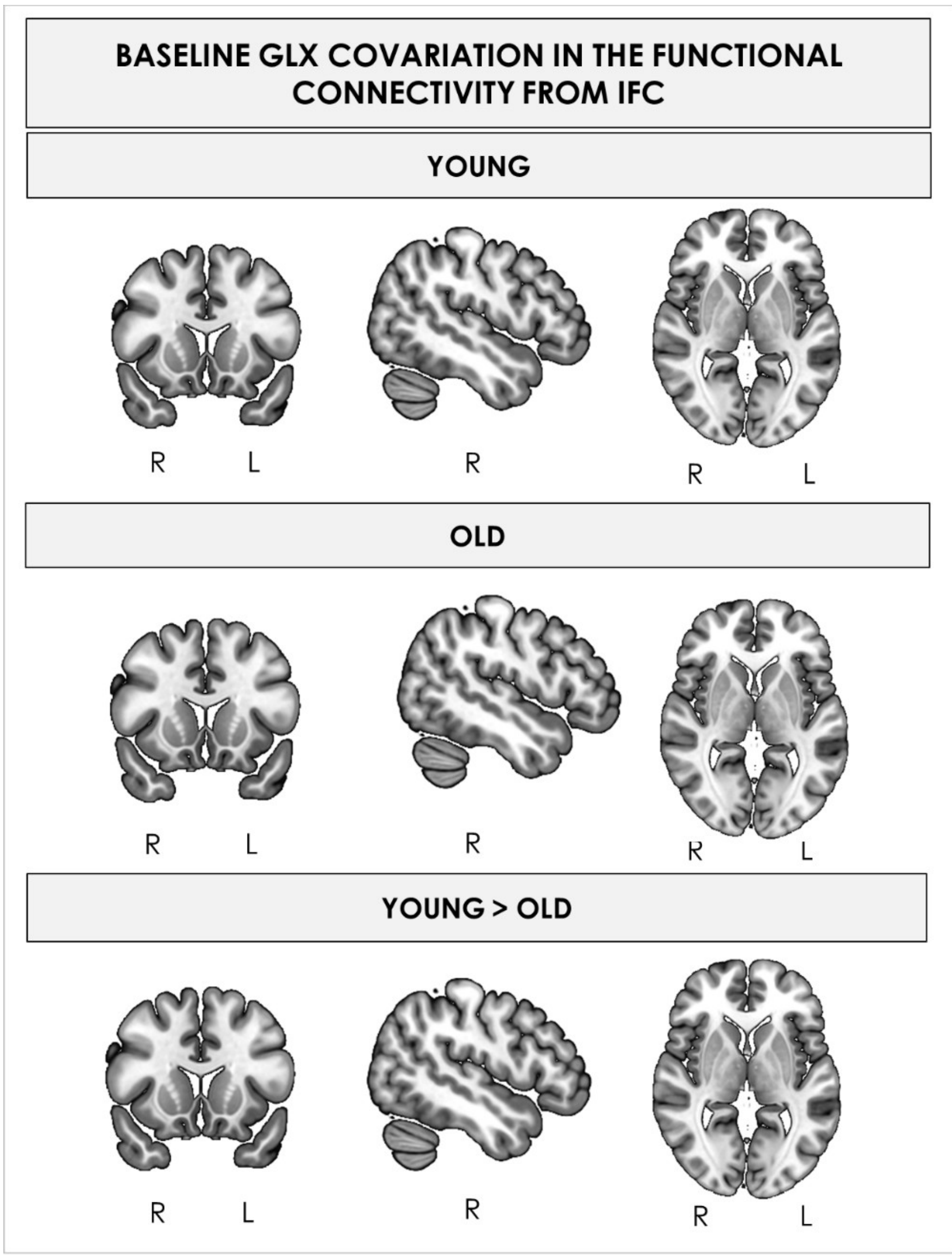

Supplementary Table 8. Association of baseline Glx in IFC with functional connectivity**.**

	Region	Z	x	y	z
Young					
	--	--	--	--	--
Old					
	--	--	--	--	--
Young > Old					
	--	--	--	--	--
Old > Young					
	--	--	--	--	--

Supplementary Figure 6. Association between GABA+ modulation and functional connectivity in the IFC (Young n=34, Old n=37). In young adults and increase in GABA+ during the task was associated with a stronger functional connectivity of IFC with other frontal regions and the supramarginal gyrus. In older adults an increase in GABA+ during the task was related with a weaker functional connectivity of IFC with several brain regions. The difference between young and older adults was significant. All maps: Pychophysiological interaction – covariation effect, z=2.7, cluster correction p=.05.

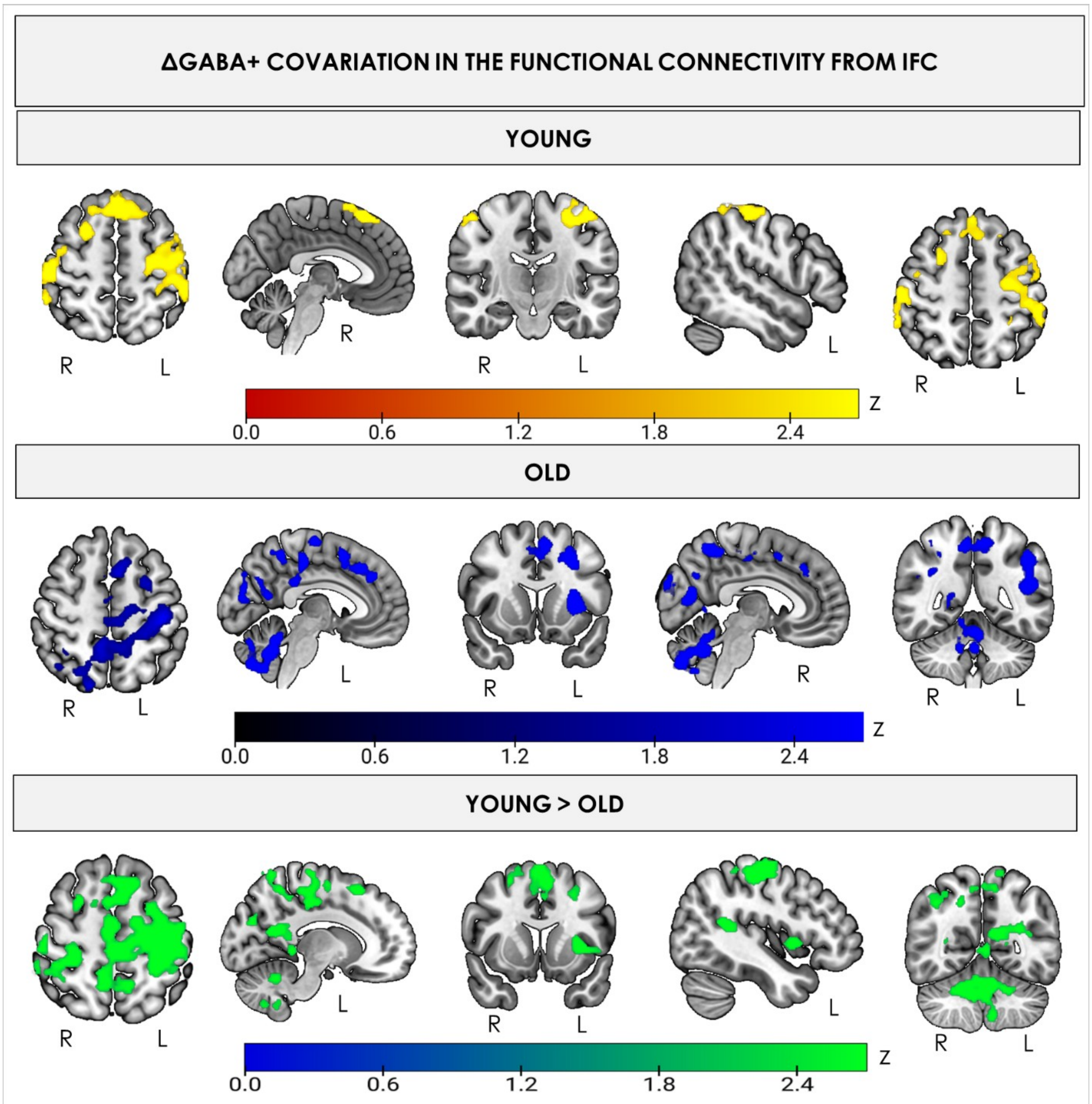

Supplementary Table 9. Association of GABA modulation in IFC with functional connectivity.

	Region	z	x	y	z
Young – Pos Effect					
	Superior Frontal Gyrus	4.27	0	32	60
	Superior Frontal Gyrus	3.97	24	8	58
	Superior Frontal Gyrus	3.59	0	16	68
	Superior Frontal Gyrus	3.53	0	12	66
	Precentral Gyrus	3.65	-46	-14	60
	Precentral gyrus	3.43	-30	-16	56
	Postcentral Gyrus	3.93	52	-24	56
	Postcentral Gyrus	2.98	-46	-30	54
	Supramarginal Gyrus	3.46	62	-42	50
	Supramarginal gyrus	3.42	-54	-34	58
	Middle Frontal Gyrus	2.91	-50	2	56
Old – Neg Effect					
	Parietal Operculum Cortex	4.06	48	-28	28
	Parietal Operculum Cortex	3.65	46	-34	30
	Postcentral Gyrus	4	-40	-26	54
	Lateral Occipital Cortex	3.44	16	-76	52
	Lateral Occipital Cortex	3.71	36	-80	20
	Lateral Occipital Cortex	3.56	22	-84	16
	Precuneus	3.4	6	-44	54
	Precuneus	3.33	8	-48	54
	Intracalcarine cortex	3.81	8	-70	12
	Cuneal cortex	3.71	-12	-78	28
	Cuneal cortex	3.67	-16	-80	24
	Occipital Pole	3.56	4	-90	24
	Supplementary Motor Cortex/Juxtapositional Lobule Cortex	3.49	-8	-18	66
	SFG	3.46	-8	12	54
	SFG	3.38	-2	38	34
	MFG	3.41	-28	12	40
	IFG, pars triangularis	3.89	-30	30	4
	Frontal Pole	3.45	-30	40	14
	Frontal Pole	2.9	-40	44	12
	Insula	4.31	-32	14	4
	Insular cortex	3.1	-38	-18	8
	Precentral Gyrus	3.39	-10	-26	46
	Supramarginal Gyrus	3.73	-60	-36	36
	Supramarginal Gyrus	3.48	-48	-48	18
	Supramarginal Gyrus	3.32	-52	-44	44
	Supramarginal Gyrus	2.86	-52	-42	32
	Angular Gyrus	3.07	-46	-52	32
	Cerebellum	4.26	0	-74	-44
	Cerebellum	4.17	0	-74	-40

	Cerebellum	3.79	26	-70	-48
	Cerebellum	3.51	-6	-60	-40
Young>Old					
	Postcentral Gyrus	4.52	-40	-26	54
	Postcentral Gyrus	3.78	-32	-36	58
	Precentral Gyrus	4.2	-26	-18	60
	Precentral Gyrus	3.89	-2	-28	54
	Supramarginal Gyrus	4.13	-46	-46	14
	Precuneus	3.86	8	-44	54
	Precuneus	3.58	-12	-54	18
	Supplementary Motor Cortex/Juxtapositional Lobule Cortex	3.82	0	8	66
	Lateral Occipital Cortex	3.56	40	-80	12
	Lateral Occipital Cortex	3.51	-32	-82	30
	Supracalcarine Cortex	3.6	-24	-60	22
	Insula	3.94	-34	14	2
	Insula	3.88	-30	22	4
	Insula	3.88	-30	28	4
	Insula	2.8	-36	-6	8
	Cerebellum	3.96	14	-54	-30
	Cerebellum	3.92	4	-76	-44
	Cerebellum	3.91	2	-76	-48
	Cerebellum	3.57	44	-78	-38
	Cerebellum	3.55	22	-58	-30
	Cerebellum	3.5	-6	-56	-28
Old>Young					
	--	--	--	--	--

Supplementary Figure 7. Association between Glx modulation and functional connectivity in the IFC (Young n=34, Old n=37). An increase in Glx was associated with a stronger functional connectivity of IFC only in older adults, where a stronger connectivity with the insula and the caudate was observed. All maps: Pychophysiological interaction – covariation effect, z=2.7, cluster

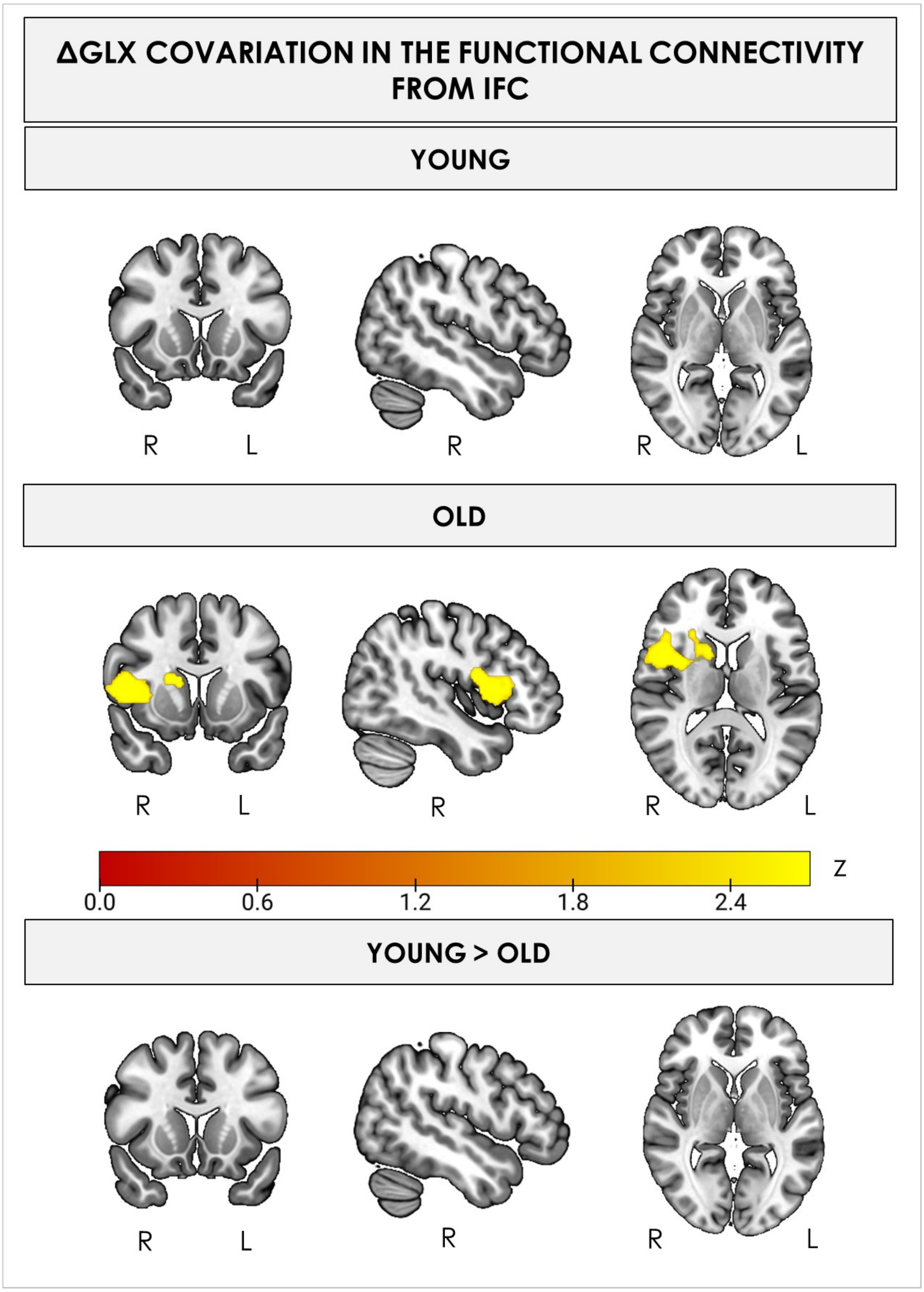

Supplementary Table 10. Association of Glx modulation in IFC with functional connectivity.

		Z	x	y	z
Young					
	--	--	--	--	--
Old Pos Eff					
	IFG pars opercularis	4.89	50	10	10
	IFG pars triangularis	2.87	44	22	8
	IFG pars triangularis	2.78	44	22	14
	Central opercular cortex	3.9	34	2	16
	Insula	3.83	38	8	4
	Right Caudate	3.31	20	10	16
Young>old					
	--	--	--	--	--
Old>Young					
	--	--	--	--	--

Supplementary Figure 8. Association between baseline GABA+ and functional connectivity in the IPL (Young n=34, Old n=39). Higher baseline levels of GABA+ in older adults were associated to a weaker functional connectivity of IPL with the frontal pole, the precuneus and the occipital cortex. No effect was observed in young adults and the difference between the two groups was significant. All maps: Pychophysiological interaction – covariation effect, z=2.7, cluster correction p=.05

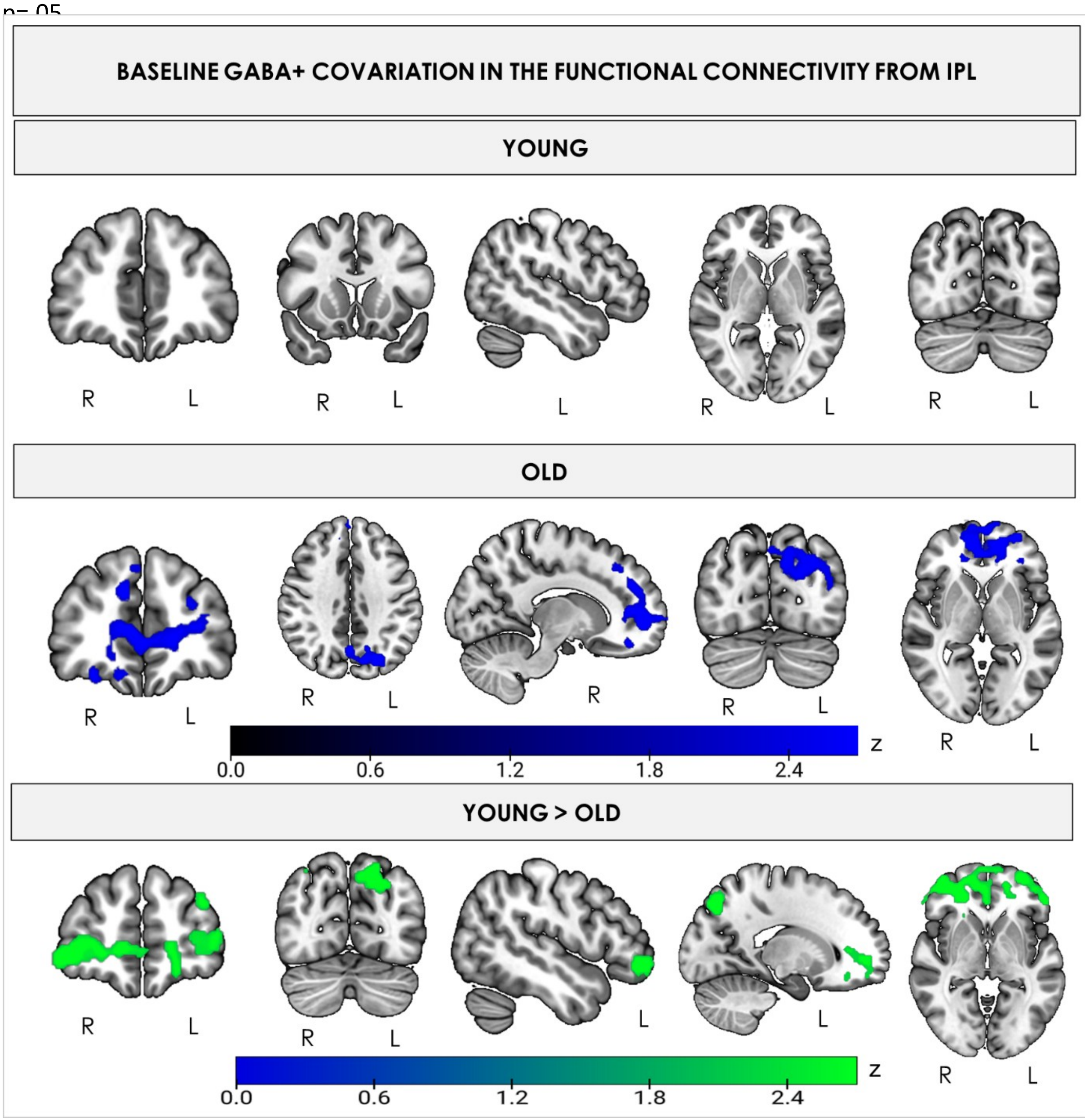

Supplementary Table 11. Association of baseline GABA+ in IPL with functional connectivity.

	Region	Z	x	y	z
Young					
	--	--	--	--	--
Old Neg Eff					
	Frontal Pole	4.02	-6	74	10
	Frontal Pole	3.75	-30	40	10
	Paracingulate Gyrus	3.73	10	54	2
	Frontal Pole	3.56	-32	58	6
	Frontal Pole	3.39	-6	68	4
	Intracalcarine Cortex	3.37	-22	-62	32
	Precuneus	3.14	-12	-76	34
	Precuneus	3.11	-14	-74	38
	Lateral Occipital Cortex	3.07	-26	-74	38
	Lateral Occipital Cortex	3.01	-24	-78	28
	Lateral Occipital Cortex	2.98	-30	-78	32
Young > Old					
	Frontal Pole	3.97	36	48	-8
	Frontal Pole	3.96	40	50	-6
	Frontal Pole	3.71	30	50	2
	Frontal Pole	3.54	52	50	-8
	Frontal Pole	3.98	-32	58	2
	Frontal Pole	3.73	-50	42	-8
	Frontal Pole	3.34	-54	38	6
	Frontal Pole	3.23	-18	52	-2
	Frontal Pole	3.17	-36	54	26
	Frontal Pole	3.09	-30	40	-10
	Lateral Occipital Cortex	3.96	-16	-74	48
	Lateral Occipital Cortex	3.21	14	-84	52
	Precuneus	3.11	-8	-62	54
	Lateral Occipital Cortex	3.11	10	-84	50
	Lateral Occipital Cortex	3.01	28	-80	50
	Lateral Occipital Cortex	2.91	22	-82	50
Old > Young					
	--	--	--	--	--

Supplementary Figure 9. Association between baseline Glx and functional connectivity in the IPL (Young n=34, Old n=39). No relation was observed between baseline Glx and the functional connectivity of IPL. However, analyses showed a minimal effect of the difference between the groups in the functional connectivity between IPL and the parietal operculum. All maps: Pychophysiological interaction – covariation effect, z=2.7, cluster correction p=.05.

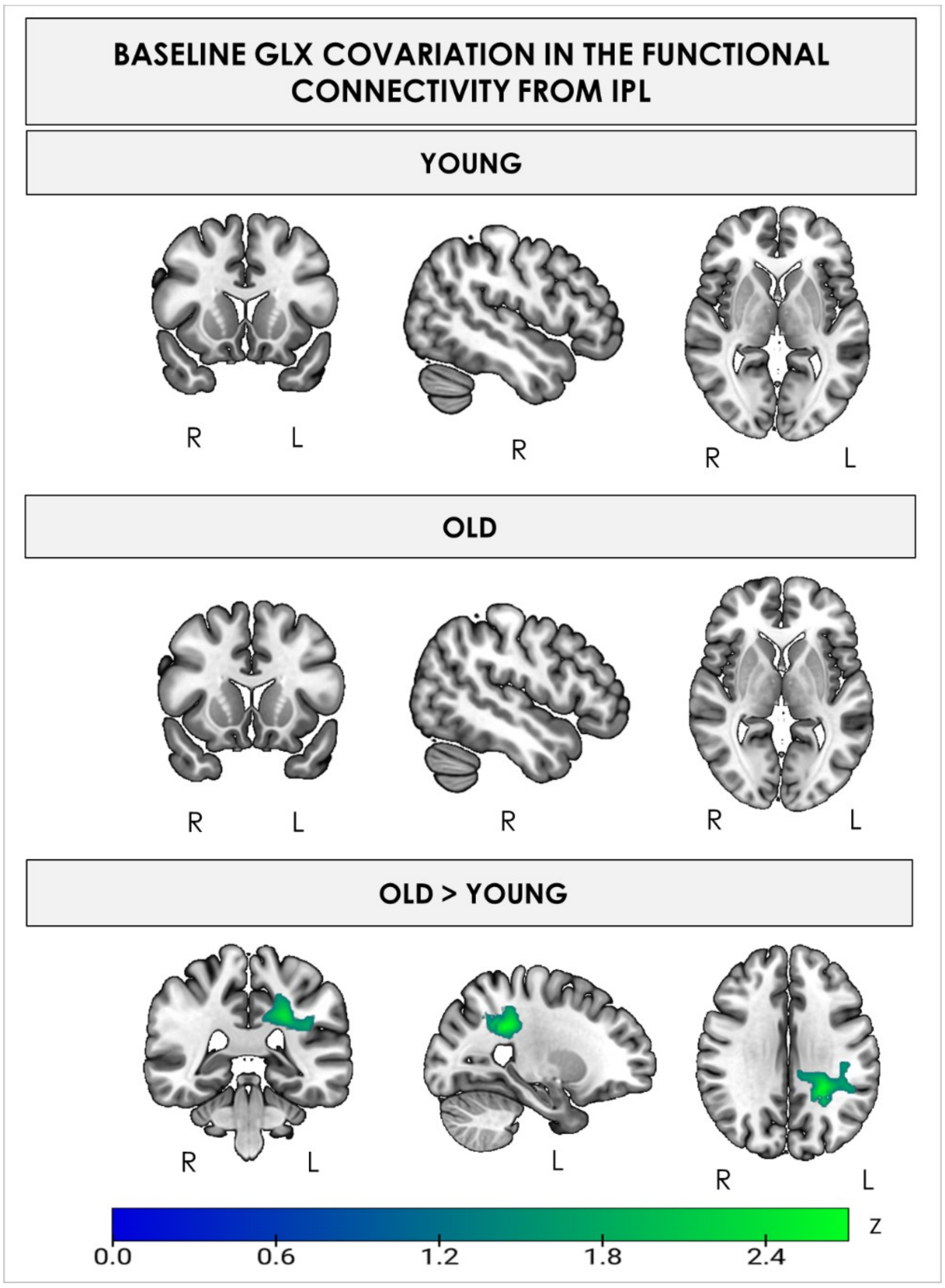

Supplementary Table 12. Association of baseline Glx in IPL with functional connectivity

	Region	Z	x	y	z
Young					
	--	--	--	--	--
Old					
	--	--	--	--	--
Young > Old					
	--	--	--	--	--
Old > Young					
	White Matter	3.43	-22	-54	38
	Parietal Operculum cortex	3.02	-42	-28	26

Supplementary Figure 10. Association between GABA+ modulation and functional connectivity in the IPL (Young n=34, Old n=39). In young adults, an increase of GABA+ was associated to a weaker functional connectivity between the IPL and the right IFC, thalamus and caudate, and the bilateral frontal pole and putamen. On the contrary, in older adults an increase of GABA+ was related with a stronger functional connectivity between the IPL and different frontal and occipital regions bilaterally. The difference between both groups was significant. All maps: Pychophysiological interaction – covariation effect, z=2.7, cluster correction p=.05.

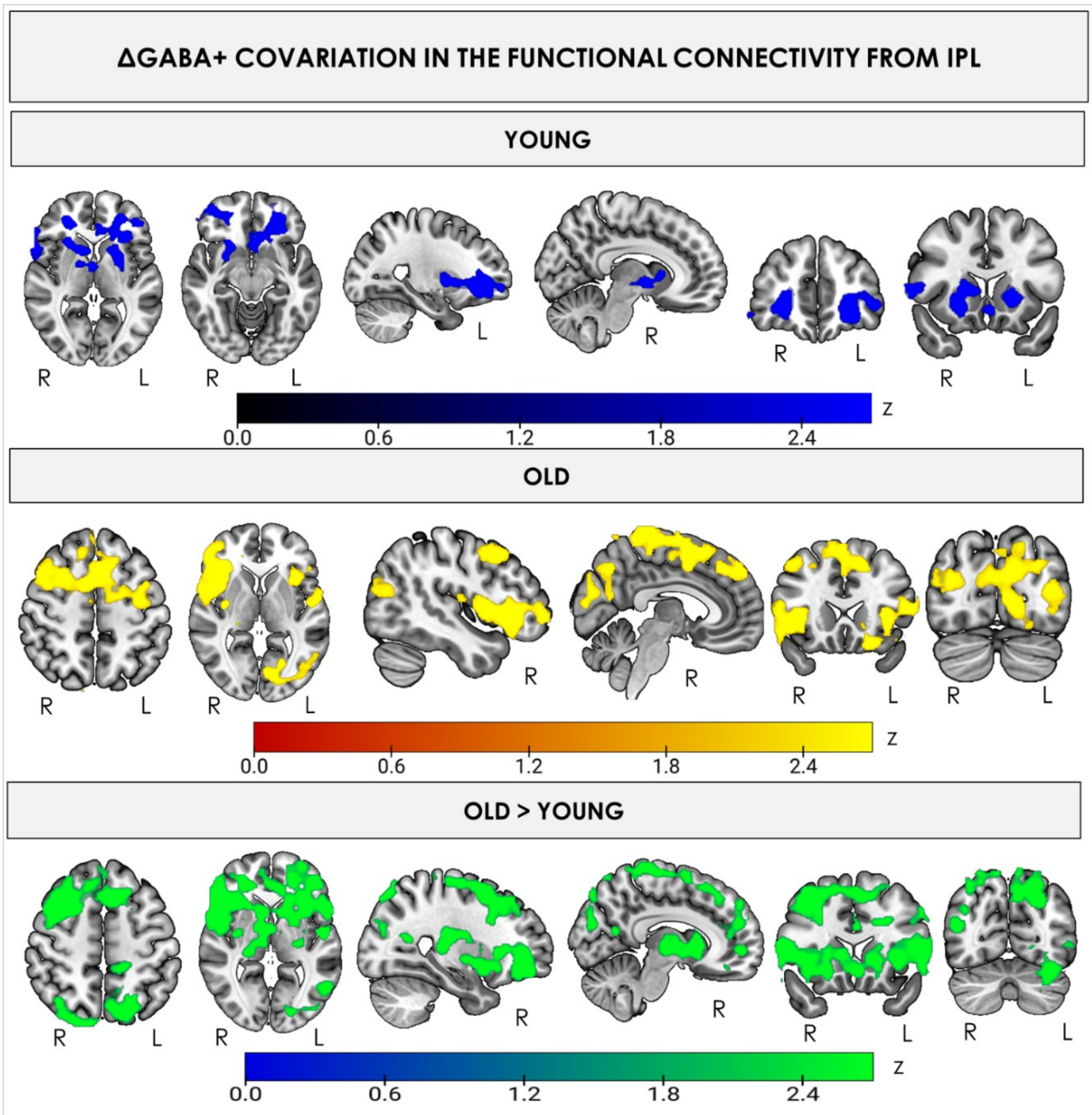

Supplementary Table 13. Association of GABA+ modulation in IPL with functional connectivity.

	Region	Z	x	y	z
Young Neg Eff					
	Frontal Pole	4.26	-30	44	-10
	Frontal Pole	3.59	38	52	-8
	Left Putamen	3.87	-22	12	-2
	Right Putamen	4.1	22	16	-6
	Right Putamen	3.37	28	4	-8
	Right Caudate	3.47	10	12	4
	Right Pallidum	3.27	16	-2	6
	Right Thalamus	4.74	2	-2	0
	Subcallosal Cortex	3.73	-4	20	-10
	IFG pars opercularis	3.96	62	14	6
	IFG pars triangularis	3.46	58	32	0
	IFG pars triangularis	3.39	60	24	2
Old Positi Effect					
	Superior Frontal Gyrus	3.87	12	0	70
	Superior Frontal Gyrus	4.05	12	-6	66
	Subcallosal Cortex	4.41	-4	12	52
	Supplementary Motor Cortex/Juxtapositional Lobule Cortex	3.99	2	8	58
	Supplementary Motor Cortex/Juxtapositional Lobule Cortex	3.78	6	4	48
	Precentral Gyrus	3.76	-32	-12	60
	Frontal Orbital Cortex	4.79	50	28	-10
	Frontal Orbital Cortex	4.7	38	24	-2
	Frontal Orbital Cortex	3.7	-24	20	-18
	Central Opercular Cortex	4.64	50	6	4
	Central Opercular Cortex	4.63	60	-2	12
	IFG pars opercularis	3.97	58	16	10
	IFG pars triangularis	3.84	-52	22	10
	IFG pars opercularis	3.8	-54	16	28
	Frontal Pole	3.76	28	38	-14
	Intracalcarine Cortex	3.96	-12	-78	2
	Intracalcarine Cortex	3.67	-22	-68	4
	Cuneal Cortex	3.8	-12	-78	28
	Lateral Occipital Cortex	3.42	34	-84	26
	Insular Cortex	4.19	-36	18	2
	Precentral Gyrus	3.76	-52	6	18
Young>Old					
	--	--	--	--	--
Old>Young					
	IFG pars opercularis	4.84	60	16	6

	Frontal Orbital Cortex	4.74	38	24	-2
	Occipital Fusiform Gyrus	4.71	-38	-66	-20
	Precentral Gyrus	4.68	62	0	12
	Cerebellum	4.57	-34	-54	40

Supplementary Figure 11. Association between Glx modulation and functional connectivity in the IPL (Young n=34, Old n=39). The changes in Glx did not hold a relationship with the functional connectivity of IPL in young adults. In older adults, an increase of glutamate related to a weaker functional connectivity of the IPL with the cerebellum and cortical areas. The difference between both age groups was significant. All maps: Pychophysiological interaction – covariation effect, z=2.7, cluster correction p=.05.

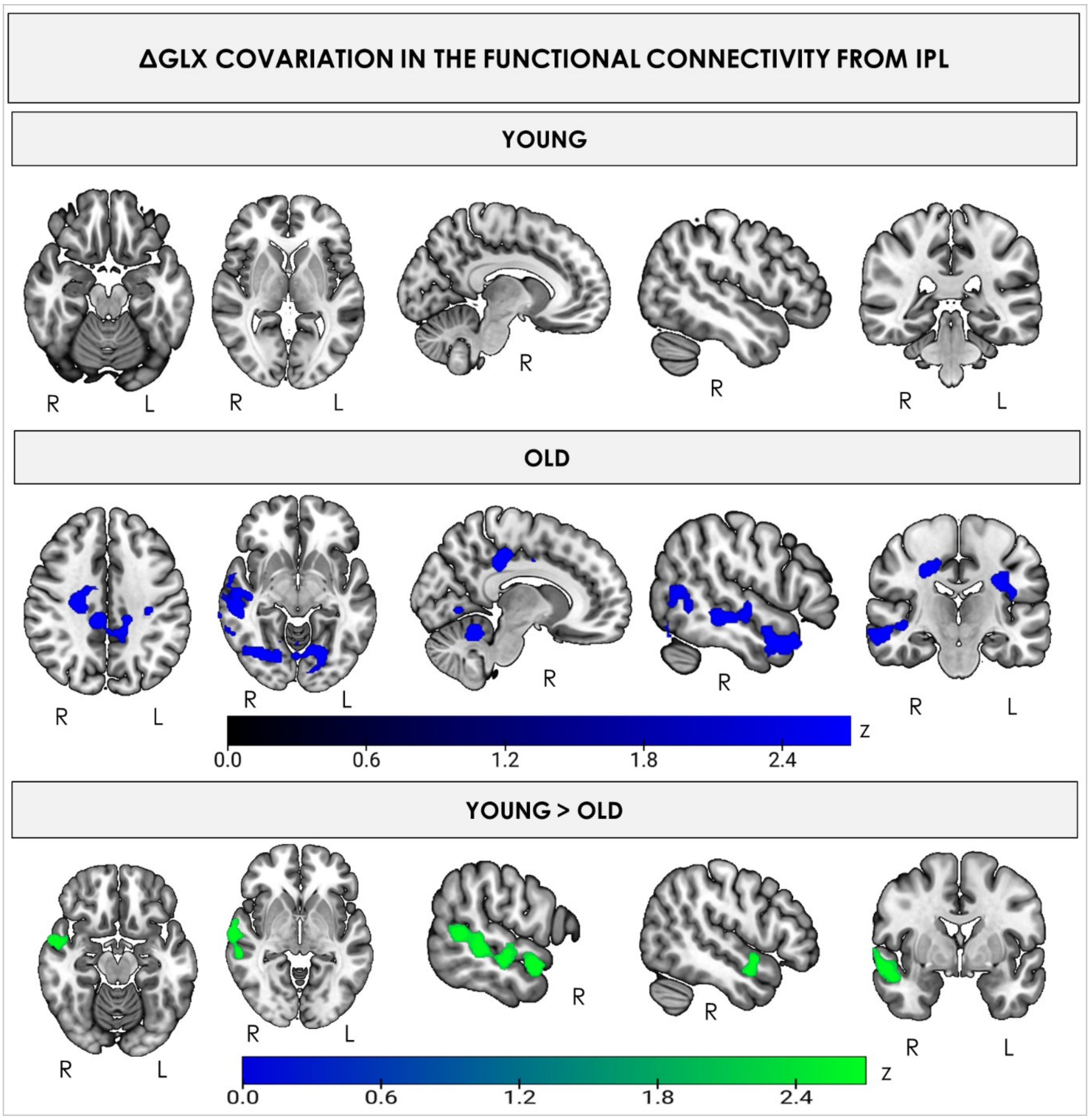

Supplementary Table 14. Association of Glx modulation in IPL with functional connectivity

	Region	Z	x	y	z
Young					
	--	--	--	--	--
Old Neg Eff					
	Cerebellum	4.64	10	-52	-20
	Cerebellum	3.62	-4	-56	-22
	Temporal Pole	3.6	50	16	-28
	Middle Temporal Gyrus	3.49	58	-18	-12
	Parietal Operculum Cortex	3.86	-40	-26	22
	Precentral Gyrus	3.75	16	-26	46
	Cingulate Gyrus	3.5	10	-32	38
Young>Old					
	Superior Temporal Gyrus	3.91	58	2	-14
	Middle Temporal Gyrus	3.4	64	-14	-2
	Middle Temporal Gyrus	3.37	62	-48	10
	Middle Temporal Gyrus	3.22	56	-16	-8

Supplementary Figure 12. Different paths in the main mediation analysis - IPL – whole brain (ΔIPL GABA+ functional connectivity - behavioral performance) in old adults.

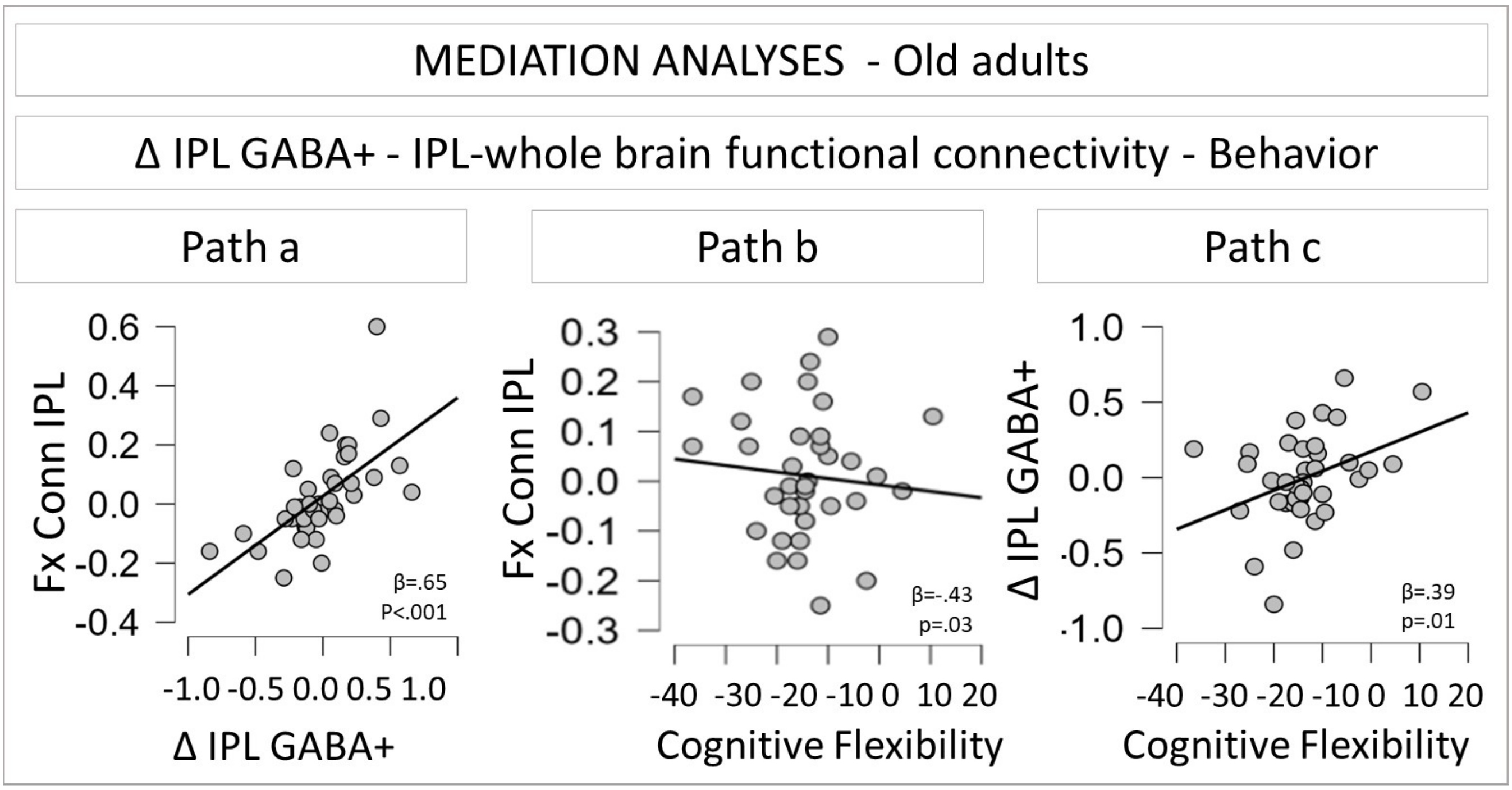

Note: The path b comprises a partial correlation (here, controlling for Δ IPL GABA+) according to mediation analyses requirements. N=39. Fx Conn – Functional Connectivity. Each datapoint represents the data from one participant. The beta coefficient represents the standardized regression weight in the mediation analyses.

Supplementary Figure 13. Different paths in the main mediation analyses (ΔIPL GABA+/Glx - IPL – region functional connectivity - behavioral performance) in old adults.

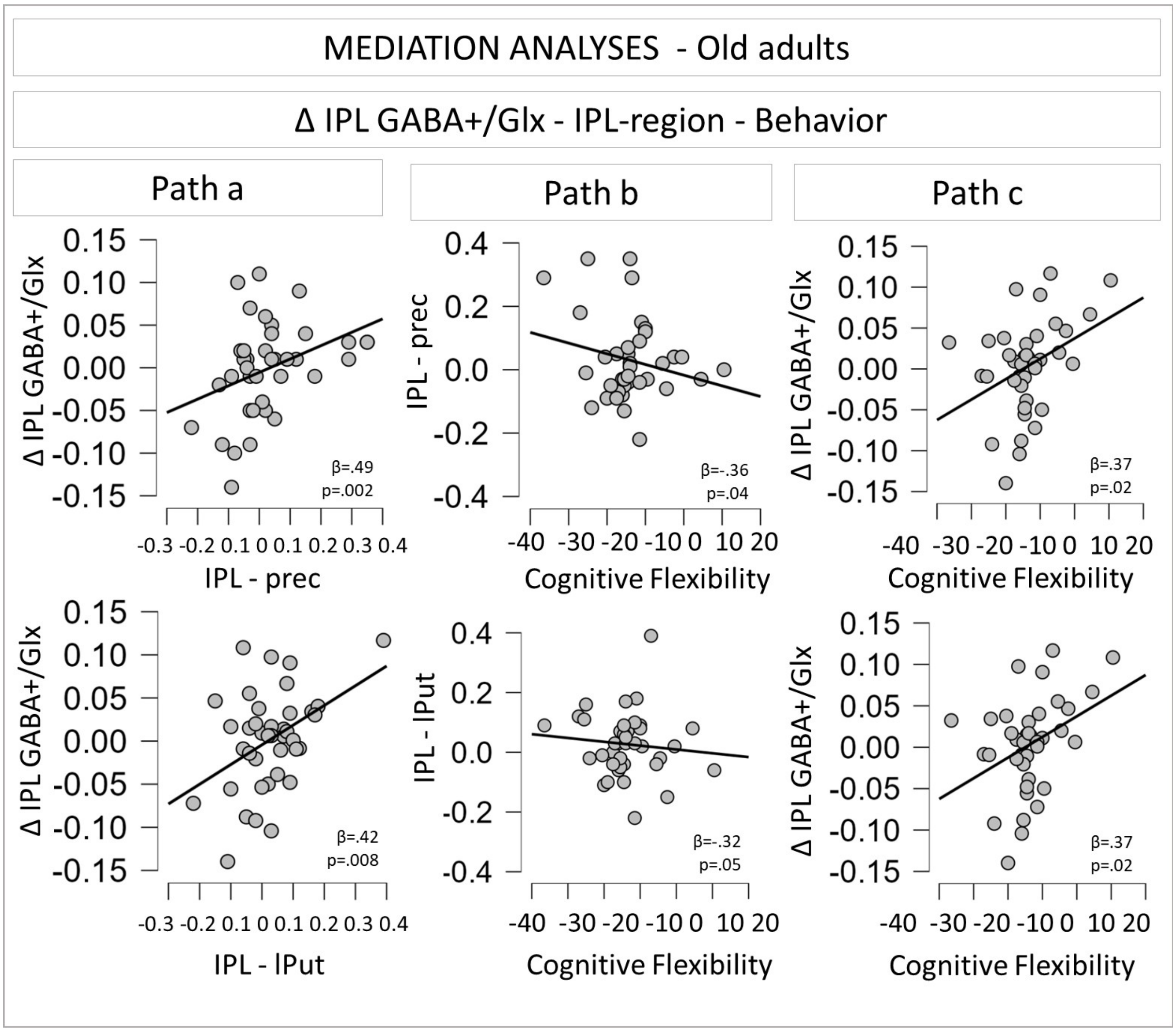

Note: The path b comprises a partial correlation (here, controlling for ΔIPL GABA+/Glx) according to mediation analyses requirements. Prec = precuneus; lPut = left putamen. N=39. Each datapoint represents the data from one participant. The beta coefficient represents the standardized regression weight in the mediation analyses.

Supplementary Figure 14. Different paths in the main mediation analyses (ΔIPL GABA+ - IPL – region functional connectivity - behavioral performance) in old adults.

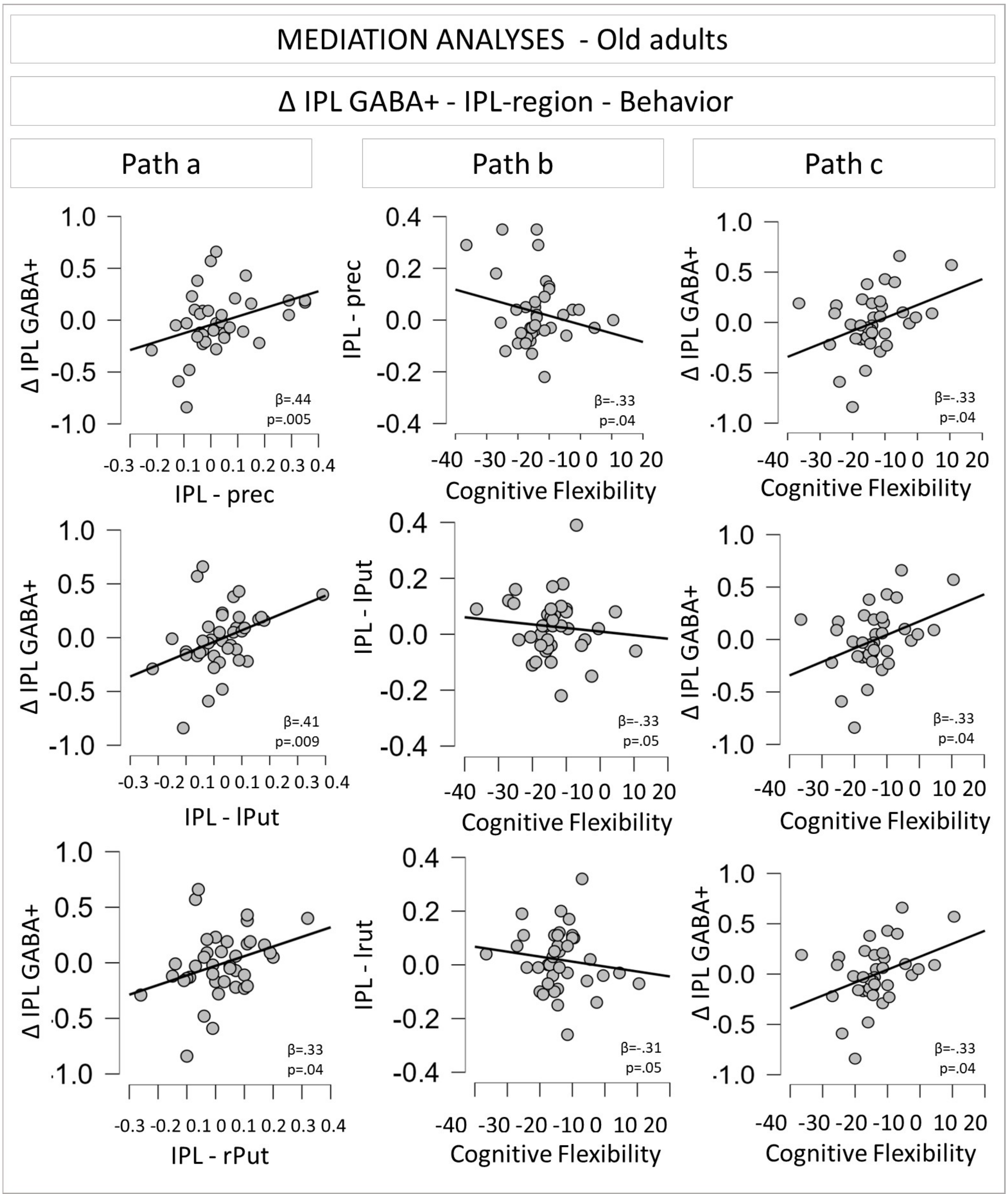

Note: The path b comprises a partial correlation (here, controlling for ΔIPL GABA+) according to mediation analyses requirements. Prec=precuneus; lPut=left putamen; rPut=right putamen. N=39. Each datapoint represents the data from one participant. The beta coefficient represents the standardized regression weight in the mediation analyses.

Supplementary Figure 15. Correlations of the different paths in the main mediation analyses (ΔIPL GABA+ - IPL – region functional connectivity - behavioral performance) in young adults.

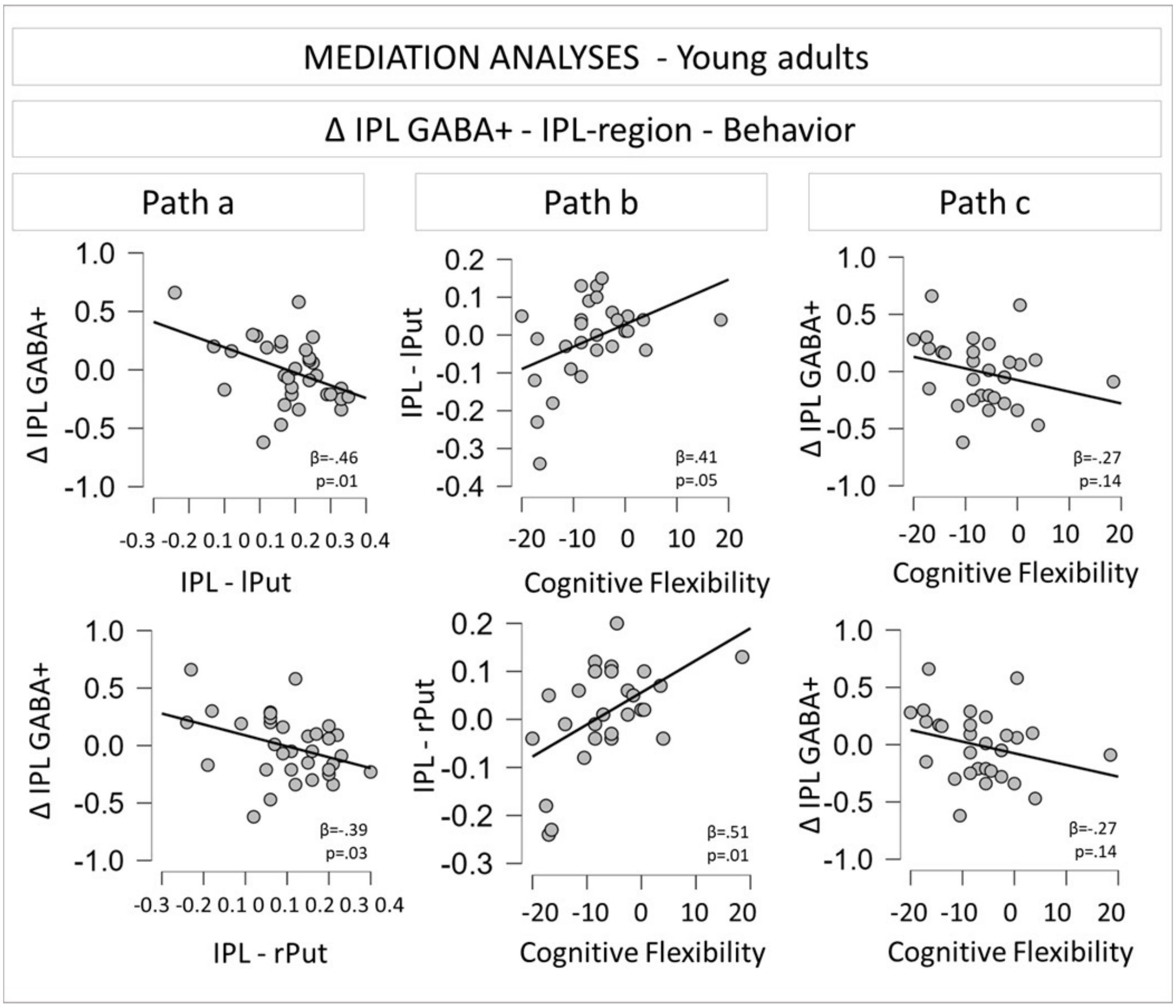

Note: The path b comprises a partial correlation (here, controlling for Δ IPL GABA+) according to mediation analyses requirements. lPut – left putamen; rPut – right putamen. N=34. Each datapoint represents the data from one participant. The beta coefficient represents the standardized regression weight in the mediation analyses.

Supplementary Table 15. Mediation analyses to assess the mediating role of functional connectivity (IPL seed; whole brain and region specific) in the relationship between metabolic modulation and behavior.

**Mediation of whole-brain functional connectivity (IPL seed)**								
**ΔGABA+/ Glx IPL**	**βc p**	**βc' p**	**βab std**	**BootSE**	**BootLLCI**	**BootULCI**	**βa p**	**βb p**
YOUNG Whole	-.36 .05	-.12 .61	-.23	.17	-.58	.14	-.73 .001	.31 .21

brain –fx conn								
OLD Whole brain-fx conn	.37<br>.02	.62<br>.003	-.24	.11	-.49	-.03	.65<br><.001	-.37<br>.06
**ΔGABA+ IPL**								
YOUNG Whole brain- fx conn	-.13<br>.56	-.28<br>.11	-.14<br>.33	-13.89	-51	.11	-.65<br><.001	.22<br>.32
OLD Whole brain- fx conn	.39<br>.01	.68<br>.001	-.28	.12	-.54	-.08	.65<br>.001	-.43<br>.03
**Mediation of IPL-brain region connectivity**								
**YOUNG ΔGABA/Glx**	**βc**<br>**p**	**βc'**<br>**p**	**βab**<br>**std**	**BootSE**	**BootLL CI**	**BootULCI**	**βa**<br>**p**	**βb**<br>**p**
IPL-IFG pt	-.36<br>.05	-.31<br>.11	-.05	.08	-.23	.11	-.29<br>.11	.16<br>.38
IPL-IFG po	-.36<br>.05	-.31<br>.11	-.05	.09	-.25	.14	-.33<br>.06	.16<br>.39
IPL-Precuneus	-.36<br>.05	-.32<br>.08	-.03	.06	-.12	.12	-.22<br>.25	.17<br>.34
IPL-Left Putamen	-.36<br>.05	-.21<br>.31	-.14	.12	-.45	.03	-.41<br>.03	.35<br>.07

IPL-Right Putamen	-.36<br>.05	-.18<br>.32	-.16	.11	-.41	.03	-.36<br>.06	.46<br>.01
**YOUNG ΔGABA**	**βc<br>p**	**βc'<br>p**	**βab<br>std**	**BootSE**	**BootLL CI**	**BootULCI**	**βa<br>p**	**βb<br>p**
IPL-IFG pt	-.27<br>.14	-.21<br>.26	-.05	.09	-.28	.11	-.29<br>.11	.19<br>.31
IPL-IFG po	-.27<br>.14	-.21<br>.28	-.06	.11	-.31	.11	-.33<br>.07	.19<br>.31
IPL-Precuneus	-.27<br>.14	-.23<br>.21	-.03	.06	-.14	.07	-.18<br>.34	.19<br>.28
IPL-Left Putamen	-.27<br>.14	-.05<br>.78	-.19	.14	-.55	.01	-.46<br>.01	.41<br>.05
IPL-Right Putamen	-.27<br>.14	-.05<br>.81	-.20	.14	-.53	.01	-.39<br>.03	.51<br>.01
**OLD ΔGABA+/Glx (IPL)**	**βc<br>p**	**βc'<br>p**	**βab<br>std**	**BootSE**	**BootLL CI**	**BootULCI**	**βa<br>p**	**βb<br>p**
IPL-IFG pt	.37<br>.02	.43<br>.01	-.06	.07	-.21	.07	.46<br>.003	-.13<br>.46
IPL-Precuneus	.37<br>.02	.55<br>.002	-.17	.09	-.38	-.002	.49<br>.002	-.36<br>.04
IPL-Left Putamen	.37<br>.02	.51<br>.003	-.13	.07	-.27	.01	.42<br>.008	-.32<br>.05
IPL-Right Putamen	.37<br>.02	.47<br>.005	-.09	.06	-.23	.02	.33<br>.04	-.29<br>.06
**OLD ΔGABA+**	**βc<br>p**	**βc'<br>p**	**βab<br>std**	**BootSE**	**BootLL CI**	**BootULCI**	**βa<br>p**	**βb<br>p**

**(IPL)**								
IPL-IFG pt	.39<br>.01	.44<br>.01	-.06	.07	-21	.07	.47<br>.002	-.13<br>.44
IPL-Precuneus	.39<br>.01	.54<br>.002	-.15	.08	-.32	.003	.44<br>.005	-.33<br>.04
IPL-Left Putamen	.39<br>.01	.53<br>.002	-.14	.07	-.28	-.007	.41<br>.009	-.33<br>.05
IPL-Right Putamen	.39<br>.01	.51<br>.002	-.11	.06	-.25	.001	.33<br>.04	-.31<br>.05

Fx conn – functional connectivity, pt – pars triangularis, po – pars opercularis
